# Cattle manure suppresses methane consumption and enhances denitrification-associated nitrous oxide production in farm dams

**DOI:** 10.1186/s40168-025-02314-4

**Published:** 2026-02-03

**Authors:** Lukas Schuster, Chris Greening, Martino E. Malerba, Stacey Trevathan-Tackett, Nadeesha Athukorala, Francesco Ricci

**Affiliations:** 1 Centre for Nature Positive Solutions, School of Science, RMIT University, Melbourne, VIC 3000 Australia; 2 School of Life and Environmental Sciences, Deakin University, Burwood, VIC 3125 Australia; 3 Department of Microbiology, Biomedicine Discovery Institute, Monash University, Clayton, VIC 3800 Australia; 4 Securing Antarctica’s Environmental Future, Monash University, Clayton, VIC 3800 Australia

**Keywords:** Community coalescence, Sustainable management, Microbial drivers, Greenhouse gas emissions, Agricultural pollution, Metagenomics

## Abstract

**Background:**

Farm dams (or agricultural ponds) are often heavily polluted freshwater systems because of nutrient-rich manure entering the water through direct deposition and runoff. Accordingly, these systems have among the highest greenhouse gas emissions per area, accounting for 41% of global freshwater methane emissions. Sustainable management actions, such as limiting livestock access through fencing, can significantly reduce nutrient concentrations and greenhouse gas emissions. However, the microbes, processes, and factors controlling greenhouse gas cycling in these systems have not been described. Here, we systematically compared the composition, functions, and activities of the microbes in paired fenced and unfenced cattle farm dams in southeastern Australia.

**Results:**

We found that in situ methane (CH_4_) and nitrous oxide (N_2_O) emissions were strongly reduced in fenced dams. Even though methanogen abundance was higher in fenced dams, fencing increased levels of aerobic methanotrophs, including two previously uncharacterised, metabolically flexible species profiled via metagenome-assembled genomes (MAGs). In contrast, we provide gene- and genome-centric evidence that N_2_O emissions are likely higher in unfenced dams due to increased production (via denitrification) rather than decreased consumption. Manure likely increases CH_4_ and N_2_O emissions primarily by driving nutrient-induced eutrophication and hypoxia that, respectively, stimulate denitrifiers and inhibit methanotrophs. However, we also provide evidence that manure-associated methanogens and bacteria occur in farm dams, where they potentially enhance emissions.

**Conclusions:**

Our findings highlight how anthropogenic activities such as livestock farming can impact microbial communities and biogeochemical cycling, thereby increasing greenhouse gas emissions from freshwater systems, and how simple management actions like fencing can mitigate such emissions.

Video Abstract

**Supplementary Information:**

The online version contains supplementary material available at 10.1186/s40168-025-02314-4.

## Introduction

In recent decades, agricultural intensification has fundamentally altered global biogeochemical cycles [1], with cascading effects on microbial communities, ecosystem functioning, and greenhouse gas emissions [2, 3]. At a global scale, agricultural systems occupy over one-third of the Earth’s surface (~ 4.8 billion hectares), with livestock farming being one of the most significant drivers of land-use change [4, 5]. When practices are unsustainable, agricultural activities can significantly contribute to eutrophication, biodiversity loss, and climate change [6]. For example, excessive nitrogen and phosphorus inputs from synthetic fertilisers and livestock manure frequently result in harmful algal blooms and the creation of low-oxygen zones in aquatic environments [7]. Elevated nutrient levels can stimulate microbial activity, accelerating organic matter decomposition and nutrient cycling, which, in turn, enhance the microbial production of potent greenhouse gases like methane (CH_4_) and nitrous oxide (N_2_O) [8]. On a 100-year timescale, CH_4_ and N_2_O are 27 and 273 times more potent at warming the climate than carbon dioxide (CO_2_), making them critical drivers of climate change [9]. Nevertheless, the ways in which biogeochemical cycling in agricultural systems contributes to climate change remain poorly understood.

Farm dams (or agricultural ponds) are some of the most heavily polluted freshwater systems as they are typically embedded within agricultural landscapes where they provide water for crop irrigation and livestock [10]. Accordingly, farm dams are subject to high levels of fertiliser runoff and the direct deposition of livestock manure, resulting in much higher nutrient levels and greenhouse gas emissions per area than other freshwater ecosystems [11–13]. In addition to nutrients, the deposition of manure may introduce gut microbiota that can persist and alter ecosystem function [14]. For example, rivers inhabited by hippos show high levels of active microbiota typically found in hippo guts, which can have profound impacts on the biogeochemical cycling in these ecosystems [15]. Similarly, manure from ruminants like cattle can enhance methanogen diversity and increase CH_4_ production in rice paddies, indicating that gut microbiota can influence carbon cycling and CH_4_ emissions in these systems [16]. Besides methanogens, cattle manure can harbour denitrifying bacteria [17], which can release N_2_O as an intermediate product of their metabolism [18]. Although the input of cattle manure can drive elevated greenhouse gas emissions from farm dams [13, 19, 20], it remains unclear whether these emissions arise from metabolic by-products of manure-derived microbes or indirectly through enhanced nutrient loading.

At a global scale, farm dams constitute less than 10% of the global lakes and freshwater ponds surface area, but they account for approximately 41% of diffusive CH_4_ emissions from global freshwater ecosystems [21]. Farm dams may also be large contributors of N_2_O emissions, with some studies reporting much higher N_2_O fluxes compared to other freshwater systems like lakes and reservoirs [22, 23], although their global contributions to anthropogenic N_2_O emissions remain unclear. Yet, simple management actions can significantly reduce nutrient loads and associated emissions from these dams. For example, Malerba et al. [19] and Odebiri et al. [20] showed that limiting livestock access to farm dams using fences reduces total nitrogen and phosphorus levels by 32–57%, along with a 56–92% reduction in CH_4_ emissions. These improvements are often accompanied by increased dissolved oxygen concentrations and decreased levels of harmful bacteria like *Escherichia coli* [24]. There are three potential mechanisms that may explain the reduction in CH_4_ emissions from fenced dams: (1) lower levels of CH_4_ production by methanogens due to reduced nutrient and substrate availability, (2) increased CH_4_ consumption by methanotrophic bacteria and/or archaea, or (3) a combination of reduced CH_4_ production and increased consumption. Similar principles may also apply to N_2_O-related dynamics, although the effects of fencing on N_2_O emissions have not yet been studied. Specifically, a reduction in nitrogen levels might limit microbial denitrification- and/or nitrification-associated N_2_O production [25], whereas increased N_2_O consumption rates driven by N_2_O-reducing bacteria (e.g. by microbes harbouring form II N_2_O reductases but not upstream denitrification enzymes) may regulate overall net emissions [26]. However, the microbial processes underlying greenhouse gas emissions remain largely unexplored, limiting our ability to predict emissions and optimise their reductions through interventions such as fencing.

In this study, we systematically evaluated the microbial communities and processes controlling greenhouse gas emissions in paired fenced vs. unfenced livestock farm dams. By integrating community, gene-centric, and genome-centric analyses with in situ CH_4_ and N_2_O gas flux measurements, we elucidate links between microbial metabolisms and observed greenhouse gas dynamics. On this basis, we explored (i) the microbes and processes responsible for CH_4_ and N_2_O emissions in farm dams, (ii) whether manure stimulates emissions through introducing nutrients or greenhouse gas-producing microbes, and (iii) whether fencing reduces production or stimulates consumption of CH_4_ and N_2_O. These findings provide a mechanistic basis to better predict and control emissions from farm dams.

## Material and methods

### Site description and experimental design

We sampled farm dams at three sites within the Yarra Ranges in Victoria, Australia, in April 2023: (1) Buttermans (37° 38′ 48″ S, 145° 19′ 44″ E), (2) Dixons (37° 35′ 56″ S, 145° 25′ 12″ E), and (3) Yarrawalla (37° 43′ 27″ S, 145° 26′ 56″ E). All sampling sites are located within 15 km of each other. The study region is characterised by a temperate climate, with an annual mean temperature of 12.1 °C and an annual mean precipitation of 419 mm (data from 1981–2022; data derived from the National Aeronautics and Space Administration (NASA) Langley Research Center (LaRC) Prediction of Worldwide Energy Resource (POWER) Project).

We sampled from two farm dams within each property, one ‘unfenced’ farm dam and one ‘fenced’ farm dam. Unfenced farm dams (*n* = 3) received no management intervention to improve their condition, with livestock freely accessing the dam. Fenced farm dams (*n* = 3) were entirely fenced off to prevent livestock access. At the time of sampling, all fenced dams had been fenced off for 10 to 15 years. All farms kept Aberdeen Angus cattle, which are 100% grass-fed. Mean cattle numbers at the different properties were ~ 200 cattle at Buttermans, ~ 20 cattle at Dixons, and ~ 300 cattle at Yarrawalla. We sampled all farm dams on the same day.

### Microbial community sampling and genomic DNA extraction

We collected three sediment and three water samples from each farm dam (3 properties × 2 dams × 2 sample types × 3 replicates = 36 samples) and three fresh (undried) cattle manure samples from the paddock of each property for microbial analyses (3 properties × 3 replicates = 9 samples). For sediment samples, we used 10-mL syringes, from which we cut off the tip to create sediment corers. We then extracted a sediment core (7 cm), from which we subsampled sediment from the lower core depths (4–7 cm sediment depth) to capture the anaerobic microbial community. For water samples, we used a 50-mL syringe fitted with a syringe filter (0.2-µm pore size) to filter water until the filter was clogged (between 8 and 50 mL of water per filter). For cattle manure samples, we used a spatula to collect fresh manure. To prevent sampling of soil, we used spatulas to pull the manure apart and sample from the upper inside layer of the pads. All samples were fixed in DNA/RNA Shield Lysis & Collection Tubes (Zymo Research International, USA) and stored at −80 °C for up to one month before DNA extractions. We also collected water quality data from each farm dam, including surface water temperature (°C), pH, and dissolved oxygen content (%) using a Hach HQ30D portable multi-meter (Hach, Australia; Table S1).

We used the ZymoBIOMICS DNA/RNA Miniprep Kit (Zymo Research International, USA) for genomic DNA extractions according to the manufacturer’s instructions. We included one negative control consisting of PCR-grade water only in each extraction round and extracted one ZymoBIOMICS Microbial Community Standard (Zymo Research International, USA) as a positive control. To verify the yield and purity of each DNA extract, we relied on spectrophotometry (NanoDrop, Nanodrop Technologies Inc., USA) and quantification by fluorometry (Qubit Fluorometer, Thermo Fisher Scientific, USA).

### Farm dam aquatic greenhouse gas flux measurements

After taking samples for microbial analysis, we deployed two floating greenhouse gas analysers known as Pondi within each dam to estimate in situ aquatic greenhouse gas fluxes. Pondi loggers consisted of a gas collection chamber (16 L in volume) fitted with a Figaro TGS2611-E00 to quantify CH_4_ levels within the chamber and a Dynament Platinum P/N2OP/NC/4/P sensor for N_2_O. Each Pondi was powered by a solar panel and battery cells and used Telstra’s Cat-M1 network to transfer data to a cloud in real-time. Full device details and specifications can be found in Malerba et al. [27]. To ensure representative flux measurements from the centre of each farm dam, we attached the sensors to a rope stretched across the dam. This setup prevented the devices from drifting and accumulating along the edges, where fluxes may be unrepresentative of open-water conditions. Each Pondi automatically recorded the concentrations of both greenhouse gases every hour for 1 week, after which we retrieved all Pondi. Floating chambers can capture constant fluxes (diffusion) and stochastic releases of gas bubbles (ebullition), which are particularly relevant for CH_4_ fluxes. Thus, both flux types were included in our greenhouse gas flux estimates.

To estimate the greenhouse gas flux of each gas, we selected segments showing a linear increase in gas concentration over time and excluded segments with signs of saturation (i.e. where concentrations plateaued) to avoid bias from saturation-induced suppression of fluxes. We then calculated the gas flux from the water surface to the atmosphere (*F*; g m^−2^ day^−1^) as:

1 $$F= \frac{\mathrm{slope} \times V \times P \times M \times {F}_{1}}{A \times R \times T \times {F}_{2}}$$

where slope is the linear rate of change in gas concentration over time within the chamber (ppm min^−1^), *V* is the chamber volume (0.01309 m^3^), *P* is the average atmospheric pressure in Pascals within the chamber during the measurement period, *M* is the molar mass of each greenhouse gas (16.04 g mol^−1^ for CH_4_ and 44.013 g mol^−1^ for N_2_O), *F*_1_ is the conversion factor from minutes to days (1440), *A* is the surface area of the chamber (0.1282 m^2^), which is equivalent to the water surface area, from which gas fluxes were measured, *R* is the ideal gas constant (8.314 J mol^−1^ K^−1^), *T* is the average temperature in Kelvin within the chamber, and *F*_2_ is the conversion factor to convert ppm (µmol mol^−1^) to mol mol^−1^ (1,000,000).

### Greenhouse gas fluxes from cattle manure incubations in the laboratory

Given that we found evidence of bacterial and archaeal taxa typically associated with cattle manure occurring in farm dams (see ‘Results’), we conducted a follow-up experiment to investigate whether these are viable in farm dams and have the potential to contribute to greenhouse gas emissions. To do so, we collected three fresh cattle manure samples at each sampling site (*n* = 9 cattle manure samples in total) in February 2024 and stored them at 4 °C overnight. The next day, we removed any dung beetles and larvae in the manure samples and subsampled ~ 30 g of manure (wet weight: 29.9 ± 0.67 g) for incubations. We also sterilised one manure sample from each sampling site in a muffle furnace at 160 °C for 2 h to use as a negative control. We placed each manure sample into separate containers (650 mL total volume). We then added 200 mL of reverse osmosis water, such that each manure sample was fully submerged, but leaving enough headspace between the water level and the container lid to fit the two greenhouse gas sensors (CH_4_ and N_2_O) of a Pondi. We sealed each chamber with parafilm to create an air-tight incubation chamber. We incubated all manure samples for 5 days at room temperature and recorded gas concentrations hourly. We then estimated greenhouse gas fluxes as described above.

### 16S rRNA gene amplicon sequencing

To infer the composition of the total bacterial and archaeal communities within each sample (*n* = 48 samples including two negative and one positive control), we used 16S rRNA gene paired-end amplicon sequencing using the universal primers 340 F [28] and 806rB [29] to target bacteria and archaea, and SSU1ArF and SSU520R to target archaea specifically [30]. All sequencing was done on an Illumina MiSeq platform at Charles River Laboratories in Melbourne, Australia.

We analysed the raw 16S rRNA gene sequences using the QIIME2 platform [31], which included quality filtering, merging, primer trimming, denoising, and rarefaction. For the taxonomic affiliation of each 16S rRNA gene amplicon sequence variant (ASV), we relied on the SILVA 138 Ref NR 99 database [32, 33]. We found that the archaea-specific primers captured an overall greater archaeal diversity than the universal primers. We thus solely focused on bacterial diversity in the data derived from the universal primers and relied on the data derived from the archaea-specific primers to map archaeal diversity in the farm dams.

We used the R package *decontam* v1.28.0 [34] to identify and remove potential contaminant ASVs from our samples based on prevalence in the negative controls. Specifically, we applied the prevalence-based method to compare the occurrence of each ASV across true samples and negative controls. This approach identified three bacterial ASVs as potential contaminants, which were subsequently removed. The contaminant sequences included ASVs assigned to *Fonsibacter* sp., an uncultured *Pseudomonas* species, and an uncultured member of the order *Bacteroidales*. Before removal, these ASVs accounted for 0.01–0.06% of the total bacterial community composition in four samples. We could not detect any archaeal contaminants.

### Microbial diversity analyses

To assess the bacterial and archaeal community structures based on the 16S rRNA gene ASV dataset, we inferred alpha and beta diversity using the R package *phyloseq* [35]. To ensure even sampling depths, we rarefied the bacterial sequences to 10,800 sequences and the archaeal sequences to 1,180 sequences. We calculated the Shannon index to assess the alpha diversity of microbial communities, whereas we measured beta diversity using the Bray–Curtis distance matrix and non-parametric multidimensional scaling ordinations (nMDS) for visualisation. To determine whether the observed between-group distances were statistically significant, we performed permutational multivariate analyses of variance (PERMANOVAs) using the ‘adonis2’ function in *vegan* [36]. In all PERMANOVA analyses, we included treatment (fenced or unfenced farm dams), sampling site (Buttermans, Dixons, or Yarrawalla), and their interaction as factors. To test for within-group multivariate homogeneity of dispersion, we performed permutational multivariate analyses of dispersion (PERMDISPs) using the ‘betadisper’ function in *vegan*. For pairwise PERMANOVAs, we used the R package *pairwiseAdonis* [37] and adjusted *P*-values using the Benjamini–Hochberg method.

### Metagenomic shotgun sequencing, assembly, and binning

We performed whole community metagenomic sequencing on a subset of samples (*n* = 12), consisting of one sediment and one water sample from each farm dam (3 fenced and 3 unfenced dams). We selected samples that exhibited the highest observed ASV richness (alpha diversity) based on 16S rRNA gene sequencing, under the assumption that these samples would best capture the overall microbial community diversity present at each site. Shotgun sequencing was conducted on an Illumina NovaSeq platform at Charles River Laboratories (Melbourne, Australia), generating 2 × 150 bp paired-end reads. Metagenomic sequencing yielded an average of 11,865,501 ± 355,837 and 6,513,684 ± 995,670 reads for the sediment and water samples from the fenced dams, respectively, and 8,387,441 ± 870,258 and 9,170,214 ± 198,840 reads for the sediment and water samples from the unfenced dams, respectively.

We used the Metaphor pipeline for read quality control, assembly, and binning of the 12 metagenomic libraries [38]. Following read quality control, we co-assembled the reads using MEGAHIT v1.2.9 [39] with default parameters. We removed any contigs shorter than 1000 bp and binned assembled contigs using VAMB v4.1.3 [40], MetaBAT v2.12.1 [41], and CONCOCT v1.1.0 [42]. We then refined the three resulting bin sets with DAS Tool v1.1.6 [43] and removed any replicates using dRep v3.4.2 [44] with 95% ANI integrated within CheckM2 [45]. To check for bin completeness and contamination, we also used CheckM2 [45]. After de-replication, we recovered 38 medium- (completeness > 50%, contamination < 10%) to high-quality (completeness > 90%, contamination < 5%) metagenome-assembled genomes (MAGs) according to the MIMAG standard [46]. We assigned MAG taxonomy according to the Genome Taxonomy Database Release R214 [47] using GTDB-Tk v2.3.2 [48], and used CoverM v0.6.1 [49] to calculate the relative abundance of MAGs based on the metagenomic short reads.

### Metabolic annotations

For community-wide metabolic analyses based on metagenomic short reads, we first stripped adapter and barcode sequences of the paired-end reads from the 12 metagenomic libraries and then used the BBDuk function of BBTools v36.92 (https://sourceforge.net/projects/bbmap/) to remove contaminating PhiX and low-quality sequences (minimum quality score of 20). We then searched the quality-filtered forward reads with lengths of at least 100 bp for the presence of the 56 marker genes representing carbon fixation, trace gas metabolism, sulfur cycling, nitrogen cycling, photosynthesis, alternative electron acceptors and donors, and aerobic respiration using the DIAMOND blastx algorithm [50]. We used a query coverage of 80% and an identity threshold of 80% for *psaA*, 75% for *hbsT*, 70% for *atpA*, *psbA*, *isoA*, *ygfK*, and *aro*, 60% for *amoA*, *mmoA*, *coxL*, *FeFe*, *nxrA*, *rbcL*, and *nuoF*, and 50% for all other genes. To calculate the proportion of community members that encoded each gene, we normalised each gene’s read count (measured in reads per kilobase million or RPKM) against the mean RPKM of 14 universal single-copy ribosomal marker genes.

For the metabolic annotation of binned and unbinned contigs, we used Prodigal v2.6.3 [51] to predict open reading frames (ORFs) and annotated them using DRAM [52] and DIAMOND BLASTP [50] homology-based searches against the Greening lab marker gene database [53], which comprises 56 metabolic marker gene sets and is continuously updated. To perform DIAMOND mapping, we used a query coverage threshold of 80% for all databases, and a percentage identity threshold of 80% for *psaA*, 75% for *mcr* and *hbsT*, 70% for *isoA*, *psbA*, *ygfK*, *aro*, and *atpA*, 60% for *amoA*, *pmoA*, *mmoA*, *coxL*, [FeFe]-hydrogenase, *nxrA*, *rbcL*, and *nuoF*, and 50% for all other databases.

To analyse the identities and capabilities of the methanotrophs present, we aligned two binned and three unbinned particulate methane monooxygenase A subunit (*pmoA*) protein sequences retrieved from our dataset with 51 reference *pmoA* protein sequences using MUSCLE [54]. We then built a maximum-likelihood phylogenetic tree using IQ-TREE v2.2.2.662 [55, 56] with 1000 ultrafast bootstraps [57] and model Q.pfam + I + R3. We plotted the tree using iTOL v6 [58] and edited it in Illustrator v24.0.2. We used the output of DRAM, which annotates contigs using KEGG [52], to provide a schematic representation of the methanotrophic MAGs classified as *Methylocystis* sp. and *CAIVXW01* sp. We used Illustrator v24.0.2 to create the schematic illustration.

### Statistical analyses

We used linear models to test for the effects of treatment (fenced or unfenced farm dams) and sampling site (Buttermans, Dixons, or Yarrawalla) on in situ farm dam and ex situ cattle manure incubation CH_4_ and N_2_O fluxes. We also used linear models to test for the effects of treatment, sampling site, and sample type (sediment or water) on the Shannon index, the relative abundances of different genera found in the farm dams, and the average copy number of genes mediating pathways of interest (methanogenesis, anaerobic and aerobic methanotrophy, nitrification-associated N_2_O production, denitrification-associated N_2_O production, and N_2_O reduction). We conducted all statistical analyses using the R package *nlme* v.3.1–168.1 [59]. When standardised residuals showed unequal variances or systematic trends, we included sampling site-, sample type, and/or treatment-specific variance coefficients in the model (function ‘varIdent’). We used the R package *emmeans* v.1.11.2 to conduct Tukey-based post hoc tests [60].

We performed all analyses and visualisations in R v4.5.0 [61]. For visualisations and plotting, we used the R packages *ggplot2* v.3.5.2 [62] and *phyloseq* v. 1.52.0 [35]. Errors reported throughout are standard errors.

## Results

### Fencing strongly but variably reduces CH_4_ and N_2_O emissions from farm dams

**Fig. 1:**
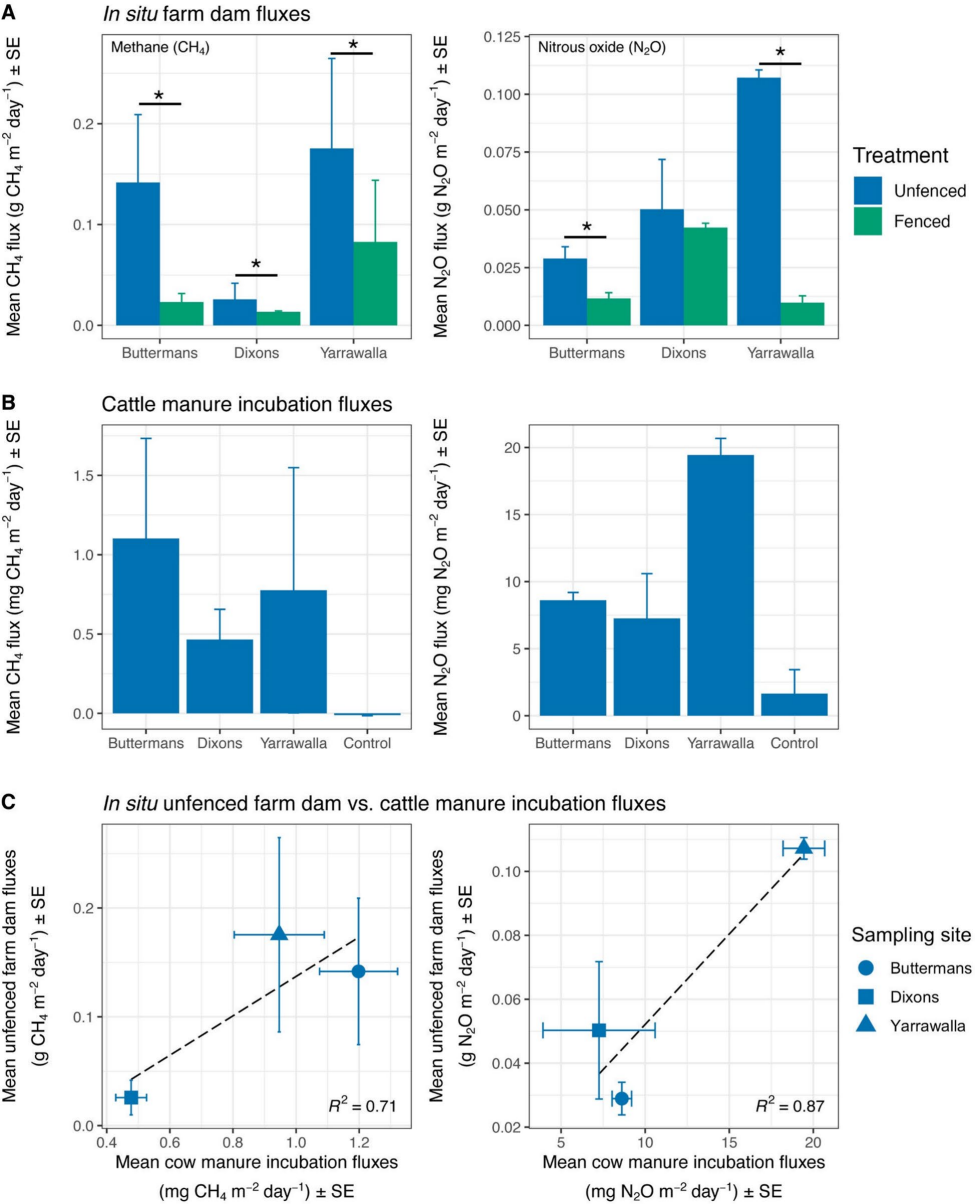
Methane and nitrous oxide emissions are lower in fenced *versus* unfenced dams. Average methane (CH_4_) and nitrous oxide (N_2_O) fluxes from **A** unfenced (blue) and fenced (green) farm dams at the different sampling sites (in g CH_4_ m^−2^ day^−1^ and g N_2_O m^−2^ day^−1^) and **B** cattle manure incubations using manure sampled at the different sampling sites (in mg CH_4_ m^−2^ day^−1^ and mg N_2_O m^−2^ day^−1^), and **C** the relationship between average fluxes from unfenced dams and average fluxes from cattle manure incubations. Error bars are standard errors

We first quantified CH_4_ and N_2_O fluxes from the different farm dams. On average, CH_4_ fluxes from the fenced farm dams were 3.5-fold lower than from the unfenced dams at all sampling sites (0.02 ± 0.008 vs. 0.07 ± 0.02 g CH_4_ m^−2^ day^−1^; *P* = 0.04; Fig. 1A; Table S2). N_2_O fluxes also differed between fenced and unfenced dams, but the magnitude of this effect varied across sampling sites (treatment × sampling site: *P* = 0.0001). At Buttermans and Yarrawalla, N_2_O fluxes were, on average, 3- and 12.2-fold lower from the fenced compared to the unfenced dams, respectively (Buttermans: 0.01 ± 0.004 vs. 0.03 ± 0.004 g N_2_O m^−2^ day^−1^; Yarrawalla: 0.009 ± 0.003 vs. 0.11 ± 0.003 g N_2_O m^−2^ day^−1^). Contrastingly, N_2_O fluxes did not significantly differ between dam treatments at the Dixons sampling site (Fig. 1A; Table S2).

We also tested whether manure causes greenhouse gas emissions through ex situ microcosm assays. All cattle manure samples produced significantly more CH_4_ and N_2_O during incubations than the sterilised control samples (CH_4_: *P* = 0.04; N_2_O: *P* = 0.001; Fig. 1B; Table S2). Furthermore, we found a positive linear relationship between greenhouse gas fluxes from cattle manure incubations and those measured in situ from unfenced dams for both CH_4_ and N_2_O (Fig. 1C).

### Greenhouse gas producers are abundant in farm dams

We next evaluated whether fencing influenced the diversity and composition of the bacterial and archaeal communities in the farm dams. Bacterial and archaeal diversities (Shannon index) significantly differed between dam treatments, but these effects were strongly site-dependent (treatment × sampling site: *P* = 0.001 for bacteria and *P* = 0.001 for archaea). Bacterial diversity was 1.1-fold higher in the fenced dam at the Dixons sampling site, whereas archaeal diversity was 1.2-fold higher in the fenced dam at Yarrawalla. Contrastingly, bacterial diversity did not significantly differ between dam treatments at Buttermans and Yarrawalla, whereas archaeal diversity was comparable between dam treatments at Buttermans and Dixons (Fig. S1; Table S3).

**Fig. 2:**
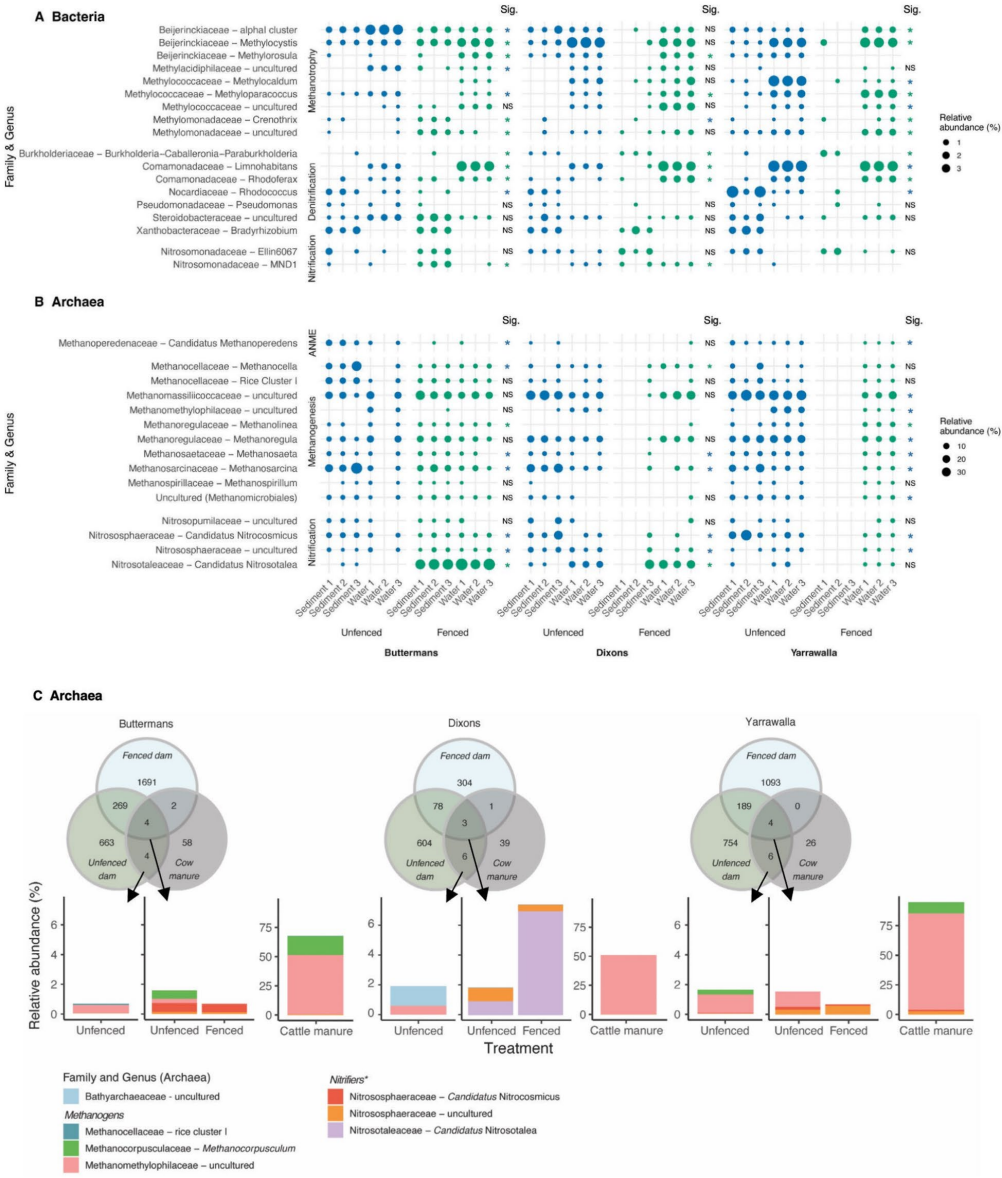
Microbes controlling greenhouse gas production differ in abundance between fenced and unfenced dams and may be introduced from manure. Relative abundances (in %) of the most abundant **A** bacterial methanotrophs, denitrifiers, and nitrifiers, and **B** archaeal anaerobic methanotrophs (ANME), methanogens, and nitrifiers found in unfenced and fenced farm dams across the different sampling sites. Bubble sizes show the relative abundances of each genus within fenced (green) and unfenced (blue) farm dams at each sampling site as a proportion of the total farm dam bacterial or archaeal communities based on 16S rRNA gene amplicon sequences. Asterisks indicate the level and direction of significant differences in relative abundance between treatments (NS = non-significant; * *P* < 0.05), with blue asterisks denoting higher relative abundance in unfenced dams and green asterisks denoting higher relative abundance in fenced dams. Genera without statistical scores had insufficient data for statistical comparisons. Statistical scores are presented in Table S6. The relative abundances of all methanotrophs, methanogens, denitrifiers, and nitrifiers found in the farm dams are shown in Fig. S3. **C** Venn diagrams and bar plots showing the numbers and relative abundances (in %) of shared archaeal ASVs between cattle manure and unfenced and fenced farm dams at the different sampling sites. Bar plots show the relative abundances of shared taxa of the total farm dam and cattle manure archaeal communities based on 16S rRNA gene amplicon sequences. The asterisk indicates taxa unlikely to originate from the cattle gut due to their obligately aerobic metabolism but likely reflect colonisation from soil or other environmental sources

Microbial community structures differed significantly between dam treatments, but these differences were not consistent across sampling sites (PERMANOVA; bacteria: *P* = 0.001; archaea: *P* = 0.001; Figs. S1 and S2; Table S4). We observed various microbes known to be capable of complete denitrification (e.g. *Paracoccus*, *Pseudomonas*, *Rhodococcus*), methanotrophy (primarily type II methanotrophs, including *Methylocystis* and members of the alphaI cluster), and nitrification (primarily uncultured Nitrosomonadaceae) (Figs. 2A and S3; Table S5). The archaeal community was dominated by methanogens, most notably *Methanosaeta* and *Methanosarcina*, which had significantly higher relative abundances in unfenced dams at all sampling sites, with some ammonia-oxidising archaea (primarily *Candidatus* Nitrocosmicus and *Candidatus* Nitrosotalea) also present (Figs. 2B and S3; Table S5).

To assess whether cattle manure may be a potential source of greenhouse gas-producing microbes in the farm dams, we compared the taxonomic profiles of manure samples with those from both fenced and unfenced dams to uncover any shared ASVs. We found that a range of microbial taxa typically found in cattle manure (Fig. S4) were also present in unfenced and fenced dams, but their relative abundances varied across sampling sites (Figs. 2C and S5). Although we could not detect any shared putative bacterial methanotrophs or denitrifiers, both fenced and unfenced dams harboured archaeal methanogens typically associated with cow manure. Specifically, shared ASVs included members of the genus *Methanocorpusculum*, uncultured members of the family Methanomethylophilaceae, and the rice cluster I group, which collectively comprised between 0.6% (Dixons) and 2.6% (Yarrawalla) of the total archaeal farm dam communities. Notably, the relative abundances of these shared methanogens in the dams were correlated with their abundances in the corresponding cattle manure at the site, where they accounted, on average, for 50.7% (Dixons) to 90.9% (Yarrawalla) of the total manure archaeal communities. These findings suggest that manure runoff or direct deposition is a major source of methanogens in the farm dams. We detected several of these manure-derived ASVs, belonging to the family Methanomethylophilaceae and the rice cluster I group, in the sediment of both fenced and unfenced dams at the Buttermans and Dixons sampling sites (0.01–0.15% of the total population), indicating that these taxa may represent established, possibly persistent populations within the dam environment. In addition to methanogens, we detected shared ASVs classified as archaeal nitrifiers in the dams, including *Candidatus* Nitrocosmicus, *Candidatus* Nitrosotalea, and uncultured members of the family Nitrososphaeraceae. However, these taxa are unlikely to originate from the cattle gut given their obligate aerobic metabolisms, and instead likely reflect colonisation from soil or other environmental sources.

### Microbial mediators of greenhouse gas fluxes from farm dams

**Fig. 3:**
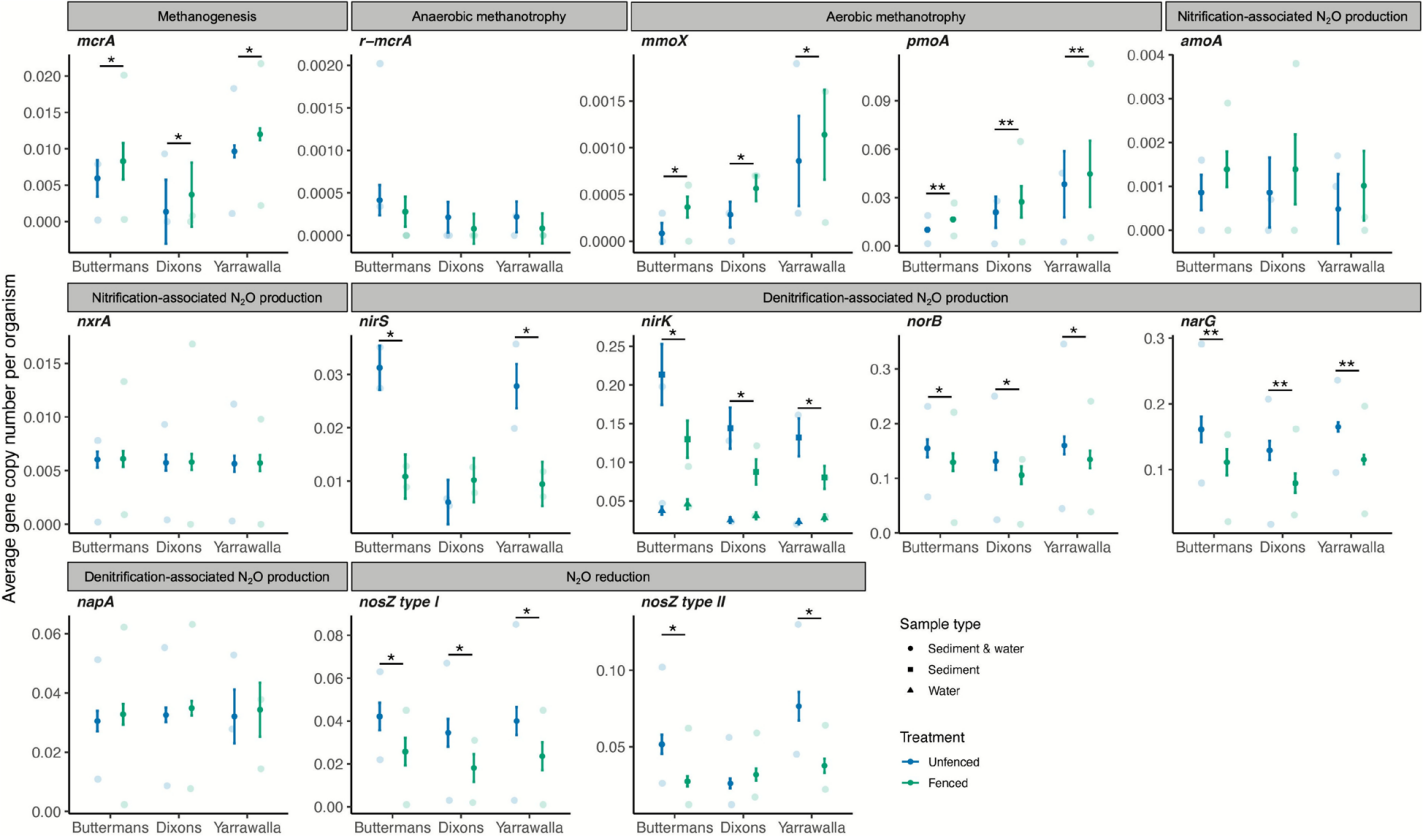
Genes controlling methane and nitrous oxide production significantly differ between fenced and unfenced dams. Average abundances of genes mediating methanogenesis (CH_4_ production), anaerobic and aerobic methanotrophy, nitrification- and denitrification-associated N_2_O production, and N_2_O reduction in unfenced (blue) and fenced (green) farm dams. Opaque points are the predicted means and are standard errors from the best-fitting statistical models; semitransparent points are the raw data; asterisks indicate significant differences

Through gene-centric metagenomic analyses, we investigated the distribution of genes mediating CH_4_ and N_2_O cycling in the water columns and sediments of the different farm dams. Average abundances of the signature gene for methanogenesis (*mcrA*; encoding the catalytic subunit of the methane-producing enzyme methyl-CoM reductase) were, on average, 1.9-fold higher in the fenced than in the unfenced dams (*P* = 0.07). However, average abundances of the genes encoding key enzymes for aerobic methanotrophy (*pmoA* and *mmoX* for particulate and soluble methane monooxygenase, respectively) were also 1.6- and 2.4-fold higher, respectively, in fenced compared to unfenced dams (*pmoA*: *P* = 0.004; *mmoX*: *P* = 0.08). We observed a significant enrichment of both methanogens and aerobic methanotrophs at all three sampling sites (Fig. 3; Table S5). In contrast, average abundances of the anaerobic methanotroph (ANME)-associated *mcrA* (*r-mcrA*) gene did not significantly differ between dam treatments (*P* = 0.26; Fig. 3; Tables S6 and S7).

Denitrification genes involved in N_2_O production differed between dam treatments. On average, the major genes for the reduction of nitrate (*narG*), nitrite (*nirK*), and nitric oxide (*norB*) were 1.5-, 1.6-, and 1.1-fold lower, respectively, in fenced than in unfenced dams (*P* = 0.001, *P* = 0.04, *P* = 0.09, respectively). Similarly, average abundances of *nirS* were threefold lower in the fenced than in the unfenced dams at Buttermans and Yarrawalla but were comparable between dam treatments at the Dixons sampling site (site × treatment: *P* = 0.05; Table S6). Contrastingly, average abundances of nitrification genes involved in N_2_O production (*amoA*, which encodes a subunit of ammonia monooxygenase, and *nxrA*, which encodes the catalytic subunit of the nitrite oxidoreductase) did not significantly differ between fenced and unfenced dams *(amoA*: *P* = 0.35; *nxrA*: *P* = 0.85*).* Finally, nitrous oxide reductase genes (*nosZ*) were lower in fenced than in unfenced dams, but the magnitude of these differences depended on gene type and sampling site. Type I *nosZ* genes typical of complete denitrifiers were 1.6-fold lower in all fenced dams, whereas type II *nosZ* genes typical of N_2_O scavengers were 1.8-fold lower in the fenced dams at Buttermans and Yarrawalla, but did not significantly differ between dam treatments at Dixons (site × treatment: *P* = 0.02). These findings are in line with the high N_2_O fluxes even in the fenced dam at the Dixons site, indicating elevated substrate availability for N_2_O reducers (Figs. 1 and 3; Tables S6 and S7). Altogether, these differences in *nosZ* gene abundances are consistent with type I-harbouring denitrifiers being less abundant in the fenced farm dams, whereas abundances of type II-carrying microbes likely reflect the variability in N_2_O production across sites. These results suggest that the increased abundances of denitrifiers, rather than lower abundances of N_2_O reducers, are responsible for enhanced N_2_O emissions in some unfenced dams.

Altogether, these patterns suggest that livestock exclusion likely shifts microbial community functions in ways that mitigate greenhouse gas emissions primarily through enhanced CH_4_ consumption and reduced denitrification-associated N_2_O production.

### Previously uncharacterised bacterial methanotrophs mitigate emissions

We next used genome-resolved metagenomics to gain a better understanding of the mediators of greenhouse gas cycling in the farm dams. Altogether, we obtained 34 high- and medium-quality metagenome-assembled genomes (MAGs). Two of these MAGs were abundant in the unfenced dams at the Buttermans and Yarrawalla sampling sites and affiliated with uncultured *Steroidobacteraceae* and *Hydrogenophaga*. Both taxa were predicted to use organic carbon, sulfide, and hydrogen as energy sources, and encode truncated denitrification pathways that can produce N_2_O (Table S8). The availability of such substrates likely results from manure runoff or direct deposition and hypoxia-associated sulfate reduction and fermentation processes in farm dams. These findings are consistent with metagenomic short-read analyses revealing a large proportion of the community can mediate fermentative hydrogen production (via group 3 [NiFe]-hydrogenases and [FeFe]-hydrogenases) and sulfide production (via dissimilatory sulfite reductase) in dam waters and sediments (Table S9). Although we did not recover any methanogen MAGs, the community structure data and *mcrA* reads both suggest that the dominant methanogens include hydrogenotrophic taxa from the family Methanomassiliicoccaceae and the genus *Methanoregula*, as well as metabolically versatile *Methanosarcina*, which can use hydrogen, acetate, and methylated compounds for methane production (Fig. 2B).

**Fig. 4:**
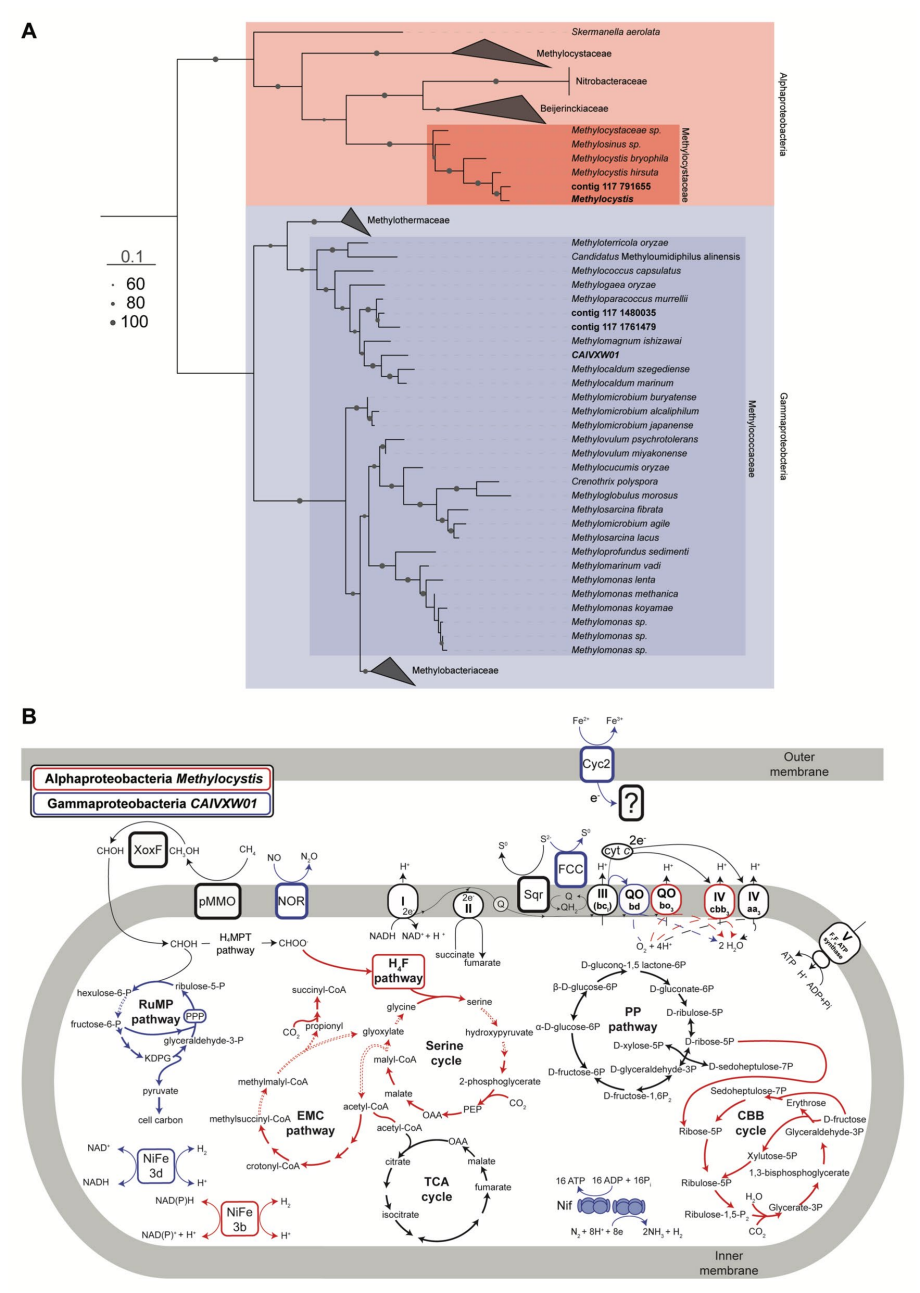
Previously uncharacterised metabolically flexible methanotrophs likely reduce methane emissions from farm dams. **A** Maximum-likelihood genome tree of two binned (*Methylocystis* sp. and *CAIVXXW01* sp.) and three unbinned (contigs in bold) PmoA proteins retrieved from our dataset with 51 reference PmoA proteins. **B** Metabolic reconstruction based on genome-resolved data of the two previously uncharacterised methanotrophs. The model depicts the inferred metabolic pathways and interactions based on the genomic content of the taxa. Red (alphaproteobacteria *Methylocystis* sp.) and blue (gammaproteobacteria *CAIVXW01* sp.) lines indicate the direction of electron acceptors and donors. Dashed arrows indicate steps where corresponding enzymes were not detected in the MAGs. The scale bar in **A** indicates the evolutionary distance, whereas dot sizes indicate bootstrap outcomes to assess the reliability of branches in the phylogenetic tree

We also identified two previously uncharacterised methanotrophic MAGs, from the alphaproteobacterial genus *Methylocystis* and candidate gammaproteobacterial genus *CAIVXW01* (family Methylococcaceae; Fig. 4A). Both MAGs were present in the water column of fenced and unfenced farm dams with similar relative abundances (0.04–0.17% of the water column microbial community reads; *P* = 0.26; Fig. S6; Table S10). Genome-resolved analyses showed that both *Methylocystis* sp. and *CAIVXW01* sp. are metabolically versatile methanotrophs capable of coupling methane oxidation to multiple redox processes. Both species encode particulate methane monooxygenase (pMMO) for methane oxidation and possess genes for methanol dehydrogenase (MDH) of the lanthanide-dependent *xoxF*-type. Furthermore, both species encode nitrogenase (NIF) for nitrogen fixation and bidirectional [NiFe]-hydrogenase group 3b in *Methylocystis* sp. and group 3 d in *CAIVXW01* sp., mediating hydrogen oxidation and production. Notably, *CAIVXW01* sp. encodes nitric oxide reductase (NOR), which catalyses the reduction of nitric oxide to N_2_O, a key intermediate in denitrification. The presence of NOR suggests that nitric oxide may serve as a terminal electron acceptor under low-oxygen conditions, enabling energy conservation through partial denitrification. Both taxa also encode sulfide:quinone oxidoreductase (SQR), with *CAIVXW01* sp. further encoding flavocytochrome *c* sulfide dehydrogenase (FCC), indicating the potential to use reduced sulfur compounds such as hydrogen sulfide (H_2_S) as electron donors. These compounds may become available at oxic-anoxic interfaces, where redox gradients are steep, particularly under hypoxic conditions. Additionally, *CAIVXW01* sp. encodes the iron-oxidising cytochrome Cyc2, suggesting a wide capacity for lithotrophy. Pathways for formaldehyde assimilation differed between the two taxa, with *Methylocystis* sp. encoding the serine cycle, whereas *CAIVXW01* sp. encoded both the ribulose monophosphate (RuMP) pathway and Calvin-Benson-Bassham (CBB) cycle genes, suggesting broader metabolic flexibility in carbon assimilation (Fig. 4B, Table S8).

## Discussion

Our study demonstrates that livestock exclusion through fencing substantially alters microbial community structure and function in farm dams, leading to lower CH_4_ and N_2_O emissions. These reductions were linked to shifts in the abundance of key microbial functional groups, including a decrease in denitrifiers and an increase in methanotrophs in fenced dams. Gene-centric analyses revealed that methanotrophs belonging to the type II subgroup were the most common methanotrophic taxa in the farm dams. Through genome-resolved metagenomic analyses, we further uncovered two previously uncharacterised methanotrophs with flexible respiratory metabolisms. In contrast, N_2_O emissions appeared to be driven by changes in denitrifier composition and function. Specifically, N_2_O production was likely associated with incomplete denitrification pathways in part of the microbial community, along with high abundances of various genera known to be capable of complete denitrification. By comparing greenhouse gas fluxes from manure incubations with in situ dam fluxes, we showed that cattle manure is a key driver of emissions in unfenced farm dams. Although we detected several methanogenic taxa found in cattle manure in the sediments of both fenced and unfenced dams, suggesting potentially persistent manure-derived methanogenic populations, their low relative abundances indicate that indigenous methanogens are likely the dominant CH_4_ producers. Altogether, our findings likely extend to other freshwater systems affected by livestock access, where similar microbial processes and nutrient-driven shifts in greenhouse gas emissions may occur.

Livestock manure can affect aquatic ecosystems by introducing nutrients and, potentially, greenhouse gas-producing microbes [8, 16]. In our study, CH_4_ and N_2_O fluxes from manure incubations were positively correlated with in situ emissions from unfenced dams, suggesting that cattle manure plays a direct and substantial role in stimulating greenhouse gas production. Consistent with these findings, in situ CH_4_ and N_2_O emissions were significantly lower in the fenced dams with no livestock access than in unfenced dams. Although the magnitude of CH_4_ emission reductions matched previous studies that investigated the effects of fencing on CH_4_ emissions [19, 20], our findings indicate that livestock exclusion through fencing may also significantly reduce N_2_O emissions from such dams. We detected several methanogenic archaea typically found in cattle manure in both fenced and unfenced dams, but we could not detect any shared denitrifiers. Although some of these manure-derived methanogens were present in the sediment in low abundances, pointing to potentially established populations [63, 64], most shared taxa were only present in the water column and, therefore, likely reflect transient inputs from deposition and runoff. These findings suggest that cattle manure likely primarily influences greenhouse gas emissions through nutrient enrichment rather than microbial seeding. Our results further indicate that herd size alone may not be a reliable indicator of emissions from farm dams, where larger herds are often associated with greater manure inputs and higher emissions. For example, the Dixons site, despite having relatively few cattle (~ 20), exhibited N_2_O fluxes comparable to the other sampling sites with much larger herds (~ 200–300 cattle). This pattern suggests that some dams may reach their greenhouse gas production capacities even at relatively low manure inputs, with increasing nutrient loadings not always resulting in increased greenhouse gas emissions. Similar threshold- or plateau-like responses to nutrient enrichment have been observed in other freshwater pond systems, where greenhouse gas production can be limited by prevailing redox conditions and other environmental factors [65, 66]. Notably, the presence of manure-associated microbes in the water column of fenced dams also suggests that manure runoff may continue to influence water quality even after livestock exclusion. If and to what extent these transient or potentially established microbial populations can influence biogeochemical cycling and long-term ecosystem function, however, remains to be studied.

Methanotrophy plays a critical role in modulating CH_4_ emissions in aquatic systems [67]. Although methanogens were more abundant in fenced dams, lower CH_4_ emissions from these dams were likely a result of increased bacterial CH_4_ oxidation, as evidenced by higher abundances of methanotroph-related genes in the fenced dams. The direct deposition and runoff of nutrient-rich manure into the farm dams likely result in fluctuating oxygen conditions typical of eutrophication [68], along with increased levels of ammonium (NH_4_^+^) and H_2_S, all of which can significantly limit methanotrophy [69, 70]. Accordingly, the previously uncharacterised methanotrophs *Methylocystis* sp. and *CAIVXW01* sp. exhibited a high degree of metabolic flexibility and likely shift to alternative energy-generating pathways, such as H_2_S-driven CO_2_ fixation or partial denitrification, when CH_4_ oxidation is inhibited. Such metabolic flexibility is well documented in methanotrophs, particularly in methanotrophic taxa belonging to the type II subgroup [71–73]. In manure-affected systems like farm dams, metabolic versatility may confer a fitness advantage by enabling adaptation to fluctuating redox conditions driven by nutrient pollution, microbial metabolisms, and seasonal variation. Altogether, these findings highlight the impacts of livestock manure on carbon cycling in unmanaged farm dams that result in significant CH_4_ emissions. Future studies should investigate whether similar mechanisms drive elevated CH_4_ emissions in other ecosystems impacted by intensive anthropogenic activity and livestock manure inputs.

High nitrogen inputs into aquatic ecosystems can stimulate microbial processes that produce N_2_O, particularly through increased rates of nitrification and denitrification [74]. In our study, several lines of evidence suggest that denitrification was the dominant pathway contributing to N_2_O emissions in farm dams, especially under unfenced conditions. We identified several taxa with complete denitrification pathways, many of which are known to accumulate N_2_O under eutrophic or low-oxygen conditions common in manure-impacted systems [75]. Consistent with these patterns, genes associated with denitrification were significantly less abundant in fenced dams across all sites, except for *nirS*, which did not differ in abundance at the Dixons site, where N_2_O emissions were similar between the fenced and unfenced dams. We also identified two MAGs with truncated denitrification pathways that were abundant in the unfenced dams with elevated N_2_O emissions but absent from the fenced dams and both dams at the Dixons sampling site. Notably, both MAGs encoded *nirS* and *norB*, enabling the reduction of nitrite to nitric oxide and subsequently to N_2_O, but lacked *nosZ*, which is required to reduce N_2_O to dinitrogen gas [76]. This genomic profile suggests that these organisms can produce N_2_O but are incapable of consuming it, thereby contributing to net N_2_O emissions. Similarly, we found that the gammaproteobacterial methanotroph *CAIVXXW01* sp. encodes *norB*, indicating its potential to couple partial denitrification with alternative carbon metabolisms to maintain energy production under conditions that limit methanotrophy. These findings highlight an intriguing intersection between CH_4_ and N_2_O dynamics, where metabolically flexible methanotrophs may directly contribute to N_2_O emissions under unfavourable conditions. Overall, our findings suggest that the composition of denitrifier communities, particularly the prevalence of *nirS*- and *nosZ*-carrying taxa, likely influences ecosystem-scale N_2_O fluxes, and highlight the role of cattle manure in shaping microbial processes that regulate agricultural greenhouse gas emissions.

Hydrogen is increasingly recognised as an ubiquitous energy source supporting microbial metabolisms in a variety of ecosystems [77]. In farm dams, hydrogen likely accumulates due to key community members shifting from aerobic respiration to hydrogenogenic fermentation following the input of manure-derived organic matter [78]. Our analyses revealed that several key microbial groups, including the previously uncharacterised methanotrophs, the two MAGs with truncated denitrification pathways, and the dominant methanogens, can use hydrogen as an electron donor. Consequently, hydrogen likely serves as a central energy currency linking carbon and nitrogen cycling in these systems. Specifically, hydrogen generated through the fermentation of organic matter likely supports methanogenesis, modulates methanotrophy under redox-variable conditions, and fuels denitrification pathways that lead to N_2_O accumulation. Together, these findings indicate that hydrogen likely plays a critical role in regulating greenhouse gas fluxes from livestock-impacted aquatic ecosystems.

## Limitations and future directions

Our study provides valuable insights into the effects of livestock manure on carbon and nitrogen cycling and associated greenhouse gas emissions from anthropogenically polluted aquatic systems like farm dams. Nevertheless, there are several limitations that warrant consideration. First, our dataset represents a snapshot in time, and seasonal dynamics in microbial activity, nutrient inputs, and greenhouse gas fluxes were not captured. Repeated sampling across hydrological and temperature gradients would help reveal temporal stability or variability in these patterns. Second, although previous studies have shown that fencing can substantially reduce nutrient concentrations (including nitrogen and phosphorus) in farm dams [19, 20], our study did not directly measure nutrient species such as dissolved organic carbon, dissolved organic nitrogen, or redox potential. This limits our ability to quantitatively link manure inputs to specific biogeochemical conditions and nutrient-driven mechanisms. Incorporating these measurements in future work will help identify the pathways underlying greenhouse gas emissions more precisely. Third, although our metagenomic analyses offer insight into the functional potential of microbial communities, they do not capture gene expression or directly quantify microbial activity. As a result, we inferred potential differences in CH_4_ and N_2_O production and consumption from the presence and relative abundance of functional genes rather than from direct rate measurements. Future studies incorporating metatranscriptomics, DNA-stable isotope probing, or assays of gross production and consumption would help confirm the activity of key taxa and more clearly disentangle the contributions of production and consumption processes to observed net fluxes. Finally, this study is limited in its geographic scope and sampling size. More extensive sampling would be required to develop more predictive insights into the physicochemical factors and microbial communities that control greenhouse gas emissions in farm dams, ideally in conjunction with structural equation modelling.

## Conclusions

Our study highlights the substantial influence of livestock manure on microbial communities mediating greenhouse gas emissions in farm dams, which are some of the most heavily impacted freshwater systems by agricultural activity. We showed that limiting livestock access through fencing significantly reduced CH_4_ and N_2_O emissions, likely due to increased abundances of methanotrophs and decreased abundances of denitrifiers. Gene-centric and genome-resolved analyses revealed that CH_4_ oxidation was primarily driven by type II and previously uncharacterised methanotrophs with metabolically flexible traits, suggesting that microbial adaptation likely plays a key role in polluted freshwater environments. Although we detected manure-associated microbes in both fenced and unfenced dams, most appeared to be transient and confined to the water column, indicating that nutrient enrichment rather than microbial seeding is the dominant driver of emissions. Nevertheless, future research should explore the impacts of manure-associated microbes on ecosystem functioning in farm dams and investigate whether similar microbial-driven greenhouse gas dynamics occur in other aquatic ecosystems that are impacted by anthropogenic activity.

## Supplementary Information

Additional file 1. Figure S1: Microbial diversities differ between fenced and unfenced dams. (A) Shannon index and (B) non-metric multidimensional scaling (NMDS) using Bray–Curtis similarity of bacterial and archaeal taxa found in the unfenced (in blue) and fenced (in green) farm dams at the different sampling sites. In (A), semi-transparent points are the raw data, opaque points are the predicted means, error bars are standard errors. Figure S2: Microbial community compositions differ between fenced and unfenced dams. Phylum-level diversity of (A) bacterial and (B) archaeal taxa in the unfenced and fenced farm dams at the different sampling sites. Relative abundances (in %) are based on 16S rRNA gene amplicon sequences. Fig. S3: Microbes controlling greenhouse gas production differ in abundance between fenced and unfenced dams. Relative abundances (in %) of (A) bacterial methanotrophs, denitrifiers, and nitrifiers, and (B) archaeal anaerobic methanotrophs (ANME), methanogens, and nitrifiers found in unfenced and fenced farm dams across the different sampling sites. Bubble sizes show the relative abundances of each genus within fenced (green) and unfenced (blue) farm dams at each sampling site as a proportion of the total farm dam bacterial or archaeal communities based on 16S rRNA gene amplicon sequences. Asterisks indicate the level and direction of significant differences in relative abundance between treatments (NS = non-significant;* *P* < 0.05), with blue asterisks denoting higher relative abundance in unfenced dams and green asterisks denoting higher relative abundance in fenced dams. Genera without statistical scores had insufficient data for statistical comparisons. Statistical scores are presented in Table S6. Figure S4: Microbes found in cow manure. Phylum and order-level diversity of (A) bacterial and (B) archaeal taxa found in cattle manure samples collected from the different sampling sites. Relative abundances (in %) are based on 16S rRNA gene amplicon sequences. Figure S5: Shared bacterial taxa between cow manure and dams differ among sampling sites. Venn diagrams and bar plots showing the numbers and relative abundances (in %) of shared bacterial taxa (based on ASVs) between cattle manure and unfenced and fenced farm dams at the different sampling sites. Bar plots show the relative abundances of shared taxa of the total farm dam and cattle manure bacterial communities based on 16S rRNA gene amplicon sequences. Fig. S6: The previously uncharacterised methanotrophs show similar abundances in fenced and unfenced dams. Relative abundances (in %) of the two previously uncharacterised methanotroph species within the gammaproteobacterial genus *CAIVXW01* and the alphaproteobacterial genus *Methylocystis* in unfenced and fenced farm dams. Opaque points are the predicted means and are standard errors from the best-fitting statistical models, semitransparent points are the raw data.

Additional file 2.

Additional file 3.

## Data Availability

All sequencing data can be found in the NCBI under the following BioProjects: 16S rRNA gene sequences (PRJNA1274188), metagenomic reads (PRJNA1273640), and MAGs (PRJNA1272758).
