# Supplementary material for: Cattle manure suppresses methane consumption and enhances denitrification-associated nitrous oxide production in farm dams
Source: Microbiome. 2026 Feb 3;14:59. doi: 10.1186/s40168-025-02314-4 (PMC12874676; doi:10.1186/s40168-025-02314-4)
Supplement: Supplementary file 2 — Additional file 1. Figure S1: Microbial diversities differ between fenced and unfenced dams. (A) Shannon index and (B) non-metric multidimensional scaling (NMDS) using Bray–Curtis similarity of bacterial and archaeal taxa found in the unfenced (in blue) and fenced (in green) farm dams at the different sampling sites. In (A), semi-transparent points are the raw data, opaque points are the predicted means, error bars are standard errors. Figure S2: Microbial community compositions differ between fenced and unfenced dams. Phylum-level diversity of (A) bacterial and (B) archaeal taxa in the unfenced and fenced farm dams at the different sampling sites. Relative abundances (in %) are based on 16S rRNA gene amplicon sequences. Fig. S3: Microbes controlling greenhouse gas production differ in abundance between fenced and unfenced dams. Relative abundances (in %) of (A) bacterial methanotrophs, denitrifiers, and nitrifiers, and (B) archaeal anaerobic methanotrophs (ANME), methanogens, and nitrifiers found in unfenced and fenced farm dams across the different sampling sites. Bubble sizes show the relative abundances of each genus within fenced (green) and unfenced (blue) farm dams at each sampling site as a proportion of the total farm dam bacterial or archaeal communities based on 16S rRNA gene amplicon sequences. Asterisks indicate the level and direction of significant differences in relative abundance between treatments (NS = non-significant;* P < 0.05), with blue asterisks denoting higher relative abundance in unfenced dams and green asterisks denoting higher relative abundance in fenced dams. Genera without statistical scores had insufficient data for statistical comparisons. Statistical scores are presented in Table S6. Figure S4: Microbes found in cow manure. Phylum and order-level diversity of (A) bacterial and (B) archaeal taxa found in cattle manure samples collected from the different sampling sites. Relative abundances (in %) are based on 16S rRNA gene amplicon [file 40168_2025_2314_MOESM1_ESM.docx]

**Supplementary materials**


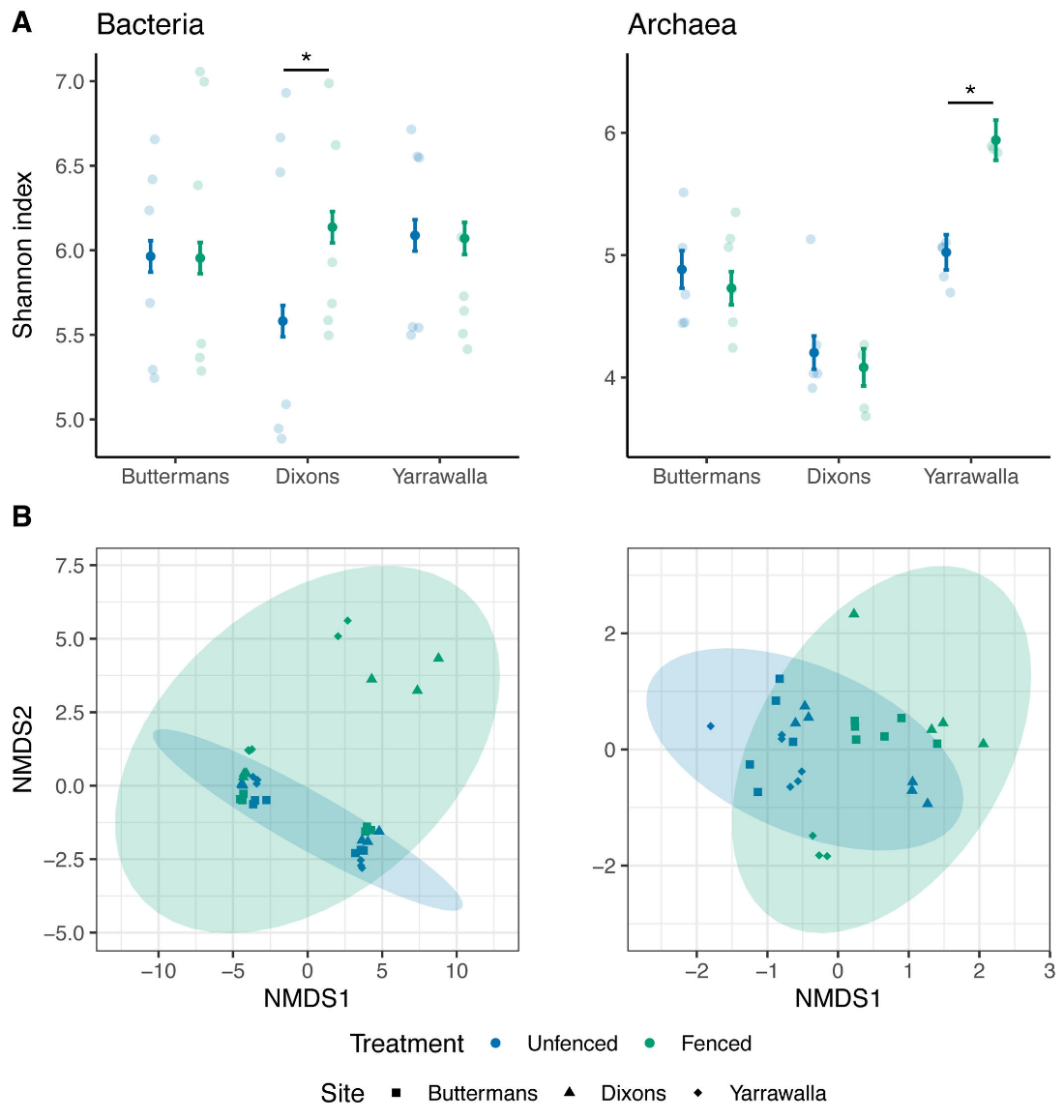


**Figure S1: Microbial diversities differ between fenced and unfenced dams.** (A) Shannon index and (B) non-metric multidimensional scaling (NMDS) using Bray-Curtis similarity of bacterial and archaeal taxa found in the unfenced (in blue) and fenced (in green) farm dams at the different sampling sites. In (A), semi-transparent points are the raw data, opaque points are the predicted means, error bars are standard errors.


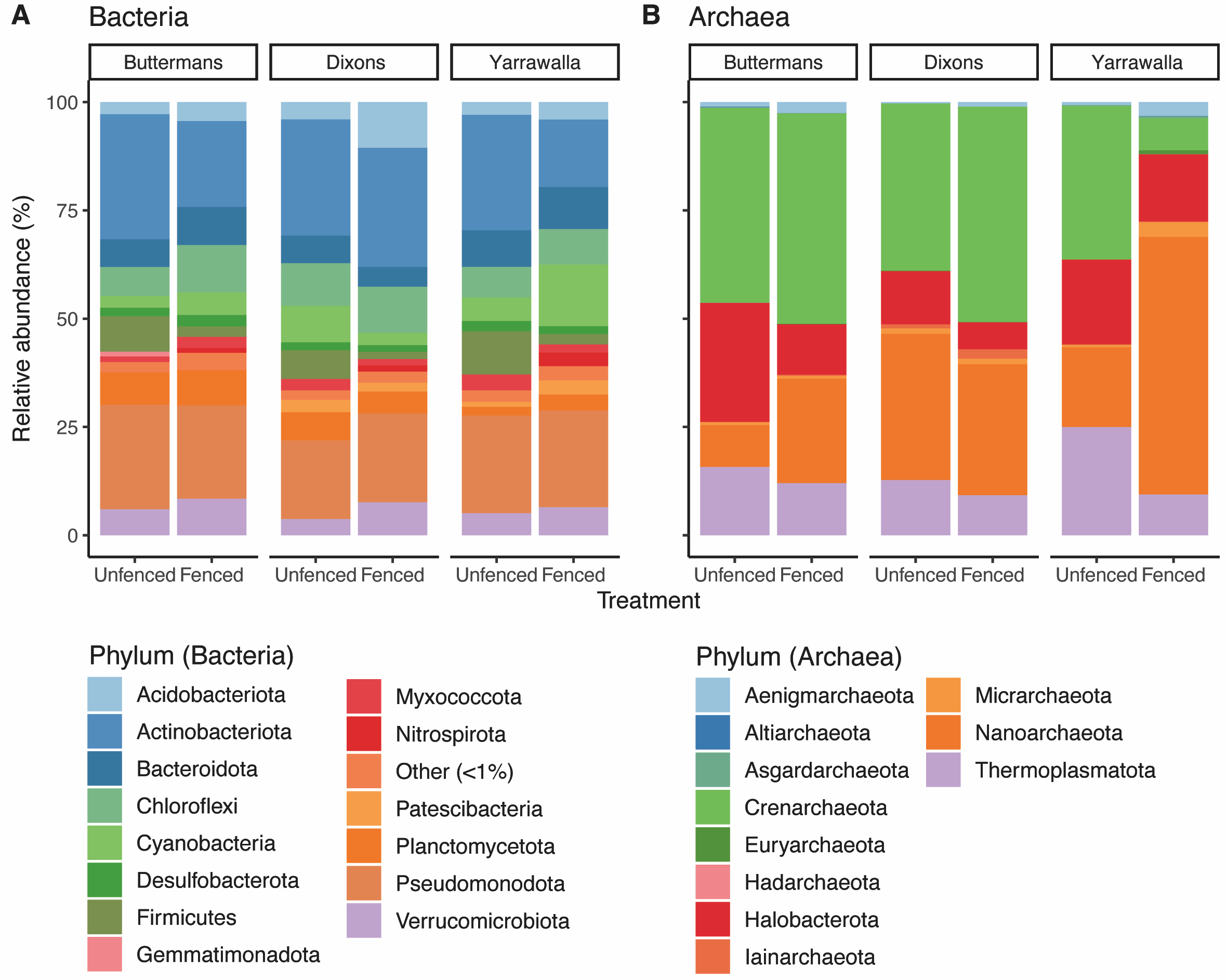


**Figure S2: Microbial community compositions differ between fenced and unfenced dams.** Phylum-level diversity of (A) bacterial and (B) archaeal taxa in the unfenced and fenced farm dams at the different sampling sites. Relative abundances (in %) are based on 16S rRNA gene amplicon sequences.

**
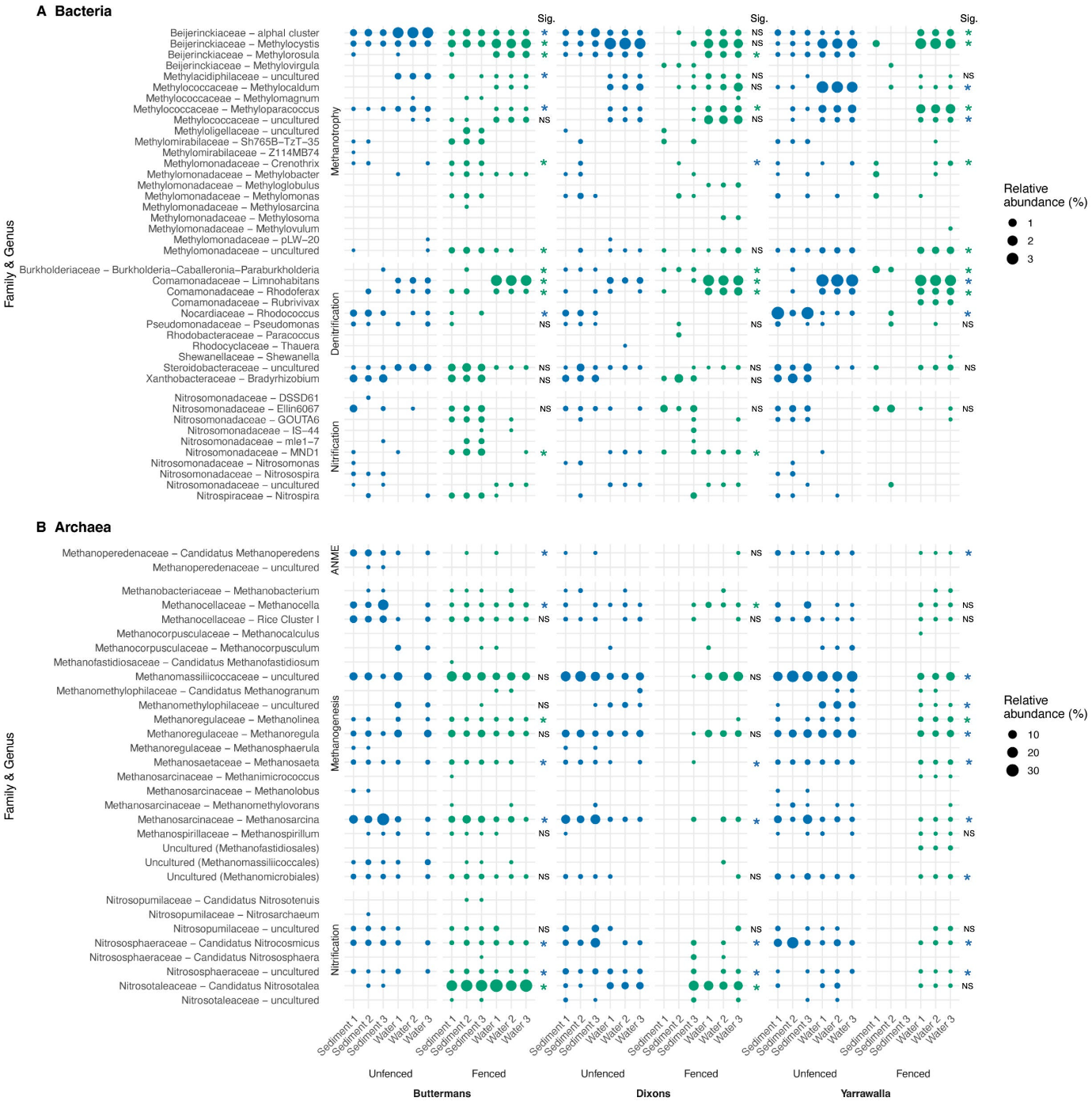
**

**Fig. S3: Microbes controlling greenhouse gas production differ in abundance between fenced and unfenced dams.** Relative abundances (in %) of (A) bacterial methanotrophs, denitrifiers, and nitrifiers, and (B) archaeal anaerobic methanotrophs (ANME), methanogens, and nitrifiers found in unfenced and fenced farm dams across the different sampling sites. Bubble sizes show the relative abundances of each genus within fenced (green) and unfenced (blue) farm dams at each sampling site as a proportion of the total farm dam bacterial or archaeal communities based on 16S rRNA gene amplicon sequences. Asterisks indicate the level and direction of significant differences in relative abundance between treatments (NS = non-significant; * *P* < 0.05), with blue asterisks denoting higher relative abundance in unfenced dams and green asterisks denoting higher relative abundance in fenced dams. Genera without statistical scores had insufficient data for statistical comparisons. Statistical scores are presented in Table S6.

**
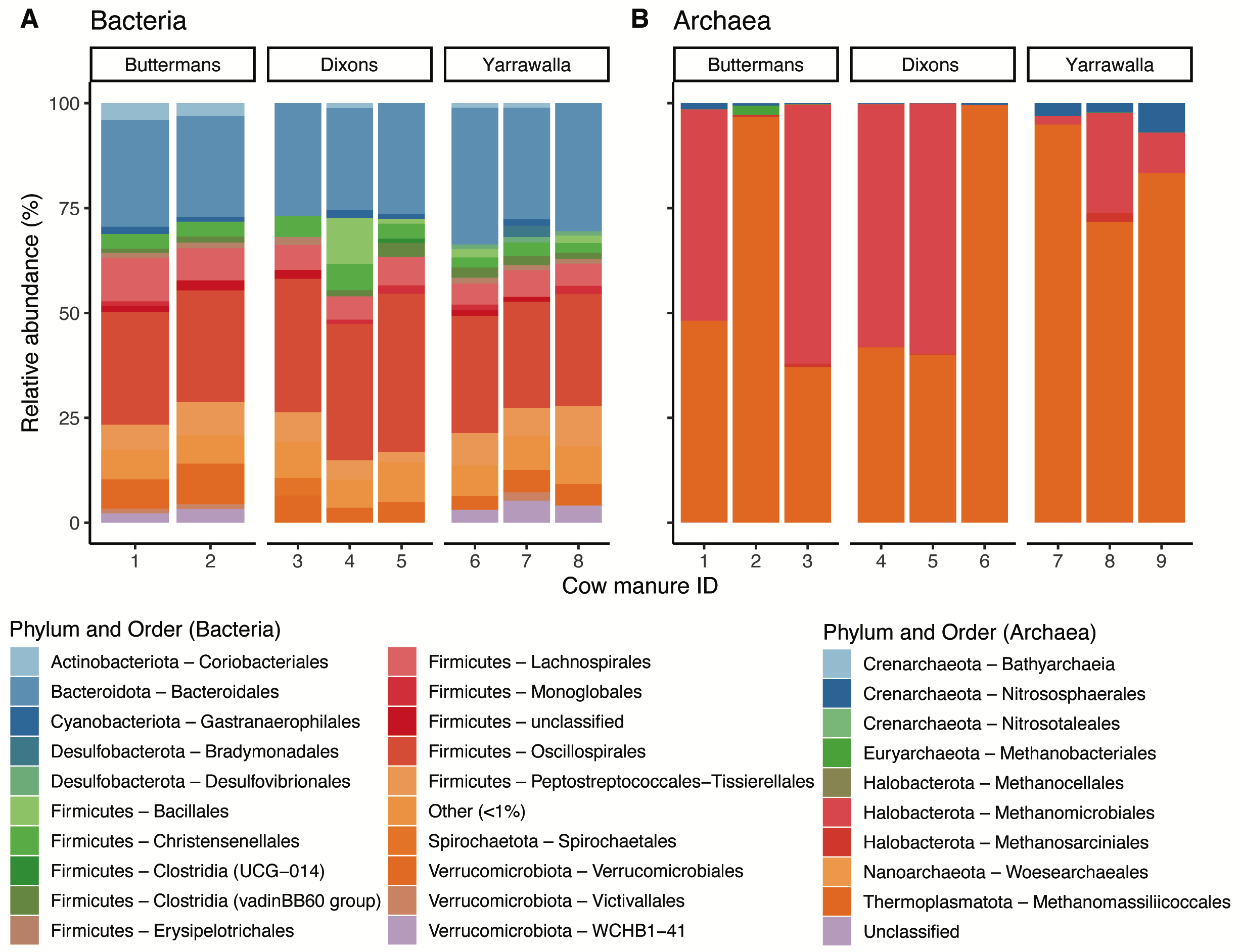
**

**Figure S4: Microbes found in cow manure.** Phylum and order-level diversity of (A) bacterial and (B) archaeal taxa found in cattle manure samples collected from the different sampling sites. Relative abundances (in %) are based on 16S rRNA gene amplicon sequences.

**
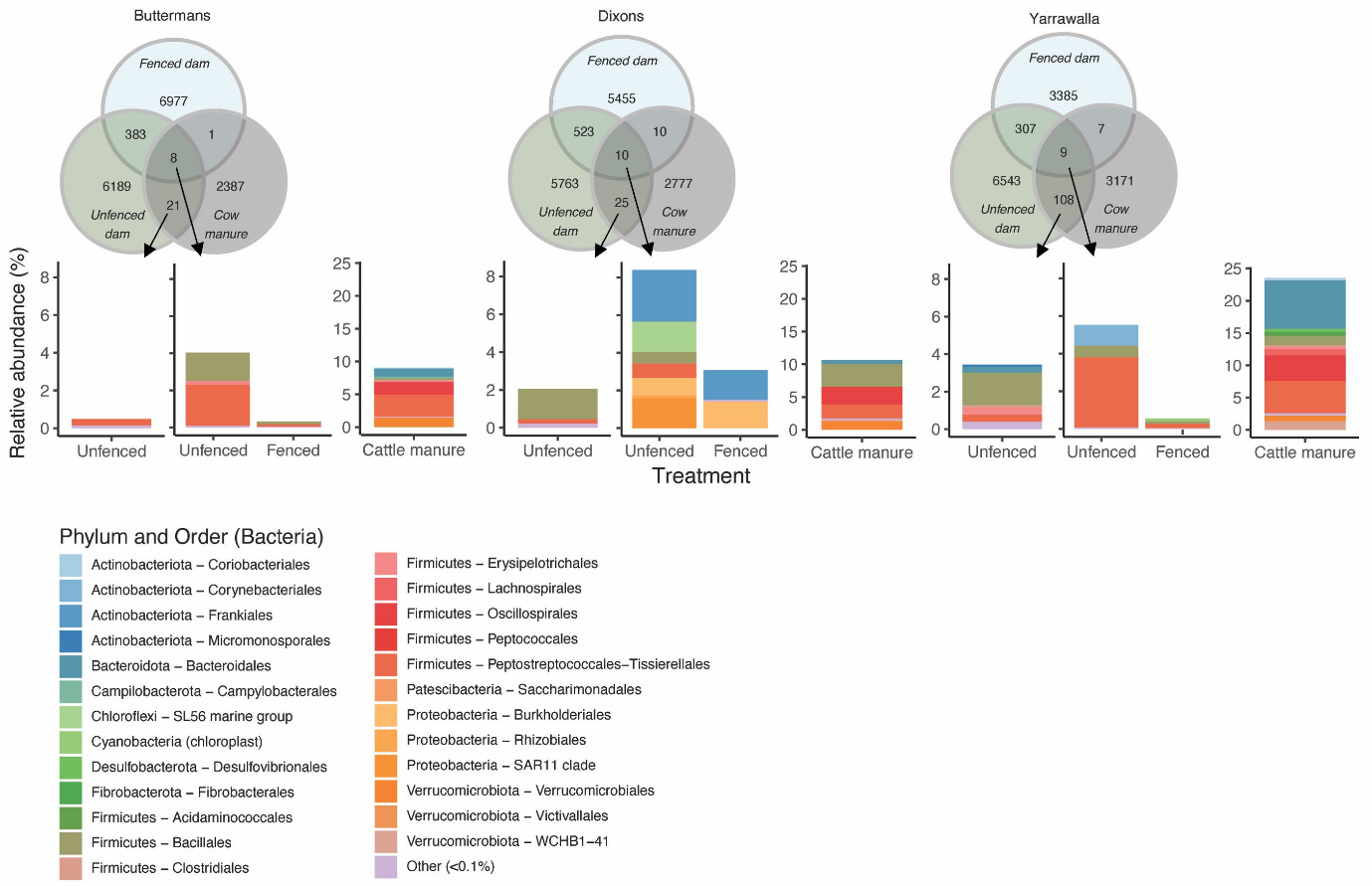
**

**Figure S5: Shared bacterial taxa between cow manure and dams differ among sampling sites.** Venn diagrams and bar plots showing the numbers and relative abundances (in %) of shared bacterial taxa (based on ASVs) between cattle manure and unfenced and fenced farm dams at the different sampling sites. Bar plots show the relative abundances of shared taxa of the total farm dam and cattle manure bacterial communities based on 16S rRNA gene amplicon sequences.

**
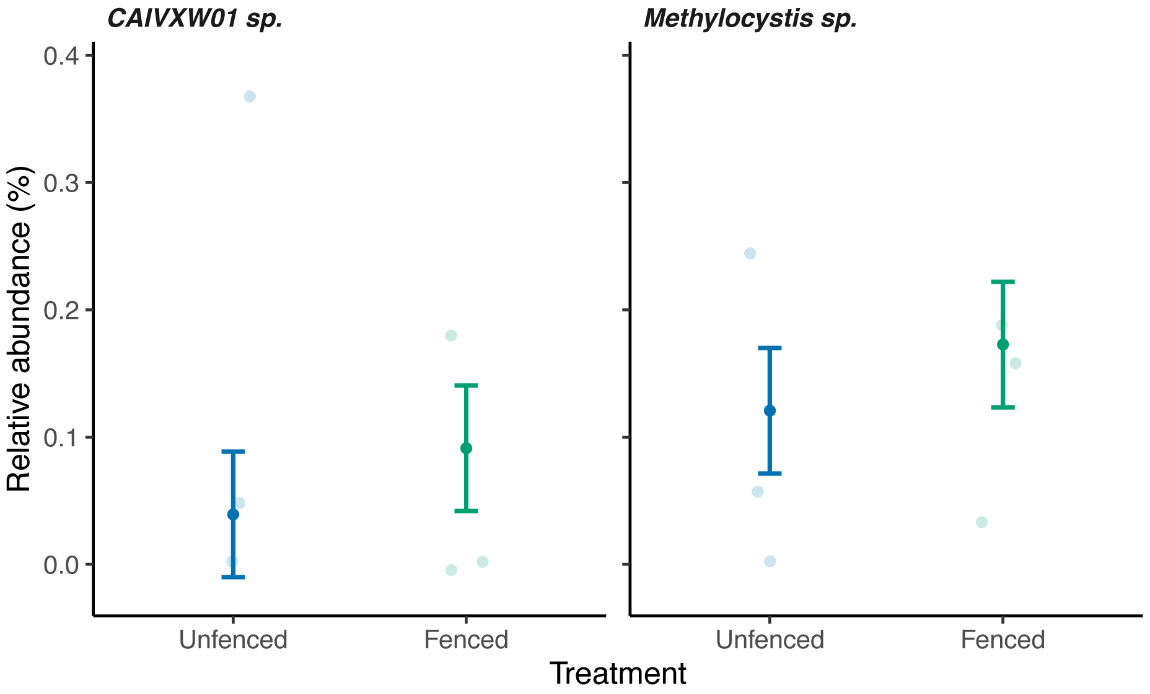
**

**Fig. S6: The previously uncharacterised methanotrophs show similar abundances in fenced and unfenced dams.** Relative abundances (in %) of the two previously uncharacterised methanotroph species within the gammaproteobacterial genus *CAIVXW01* and the alphaproteobacterial genus *Methylocystis* in unfenced and fenced farm dams. Opaque points are the predicted means and are standard errors from the best-fitting statistical models, semitransparent points are the raw data.

**Table S1:** Water quality parameters of the unfenced and fenced farm dams at the different sampling sites.

| **Sampling site** | **Treatment** | **Dissolved oxygen (%)** | **pH** | **Surface water temperature (ºC)** |
| --- | --- | --- | --- | --- |
| Buttermans | Unfenced dam | 99^*^ | 8.57 | 21.2 |
|  | Fenced dam | 43 | 6.83 | 20.5 |
| Dixons | Unfenced dam | 23.3 | 7.35 | 13.1 |
|  | Fenced dam | 13 | 6.82 | 15.7 |
| Yarrawalla | Unfenced dam | 20.7 | 7.05 | 16.7 |
|  | Fenced dam | 24 | 7 | 17.6 |

^*^Algal bloom observed at time of sampling.

**Table S2:** Outcome of linear models testing for the effects of treatment (fenced or unfenced farm dam) and sampling site on (A) *in situ* farm dam fluxes, (B) cattle manure incubation fluxes, and (C) the relationship between average unfenced *in situ* farm dam fluxes and cattle manure incubation fluxes. DF are degrees of freedom.

|  | **Numerator DF** | **Denominator DF** | ***F* value** | ***P* value** |
| --- | --- | --- | --- | --- |
| 1. ***In situ* farm dam fluxes** |  |  |  |  |
| 1. **Methane (CH_4_) fluxes** |  |  |  |  |
| Intercept | 1 | 8 | 34.05 | 0.0004 |
| Treatment | 1 | 8 | 6.17 | 0.04 |
| Sampling site | 2 | 8 | 4.58 | 0.04 |
| 1. **Nitrous oxide (N_2_O) fluxes** |  |  |  |  |
| Intercept | 1 | 6 | 52.3 | 0.0004 |
| Treatment | 2 | 6 | 9.31 | 0.03 |
| Sampling site | 1 | 6 | 118.97 | <0.0001 |
| Treatment × Sampling site | 2 | 6 | 65.16 | 0.0001 |
| 1. **Cattle manure incubation fluxes** |  |  |  |  |
| 1. **Methane (CH_4_) fluxes** |  |  |  |  |
| Intercept | 1 | 5 | 6.15 | 0.05 |
| Sampling site (including control) | 3 | 5 | 5.77 | 0.04 |
| 1. **Nitrous oxide (N_2_O) fluxes** |  |  |  |  |
| Intercept | 1 | 5 | 220.12 | <0.0001 |
| Sampling site (including control) | 3 | 5 | 28.77 | 0.001 |

**Table S3:** Outcome of linear models testing for the effects of treatment (fenced or unfenced farm dam), sampling site, and sample type (sediment or water) on (A) bacterial and (B) archaeal Shannon indices. DF are degrees of freedom.

|  | **Numerator DF** | **Denominator DF** | ***F* value** | ***P* value** |
| --- | --- | --- | --- | --- |
| 1. **Bacteria** |  |  |  |  |
| Intercept | 1 | 28 | 2297.33 | <0.0001 |
| Treatment | 1 | 28 | 0.01 | 0.93 |
| Site | 2 | 28 | 11.77 | 0.001 |
| Sample type | 1 | 28 | 85.68 | <0.0001 |
| Site × treatment | 2 | 28 | 9.09 | 0.001 |
| 1. **Archaea** |  |  |  |  |
| Intercept | 1 | 22 | 700.04 | <0.0001 |
| Treatment | 1 | 22 | 0.6 | 0.45 |
| Sample type | 1 | 22 | 0.22 | 0.65 |
| Site × treatment | 1 | 22 | 9.74 | 0.001 |

**Table S4:** Outcome of permutational multivariate analyses of variance (PERMANOVAs) testing for the effects of treatment (fenced or unfenced farm dam), sample type (water or sediment) and sampling site on (A) bacterial and (B) archaeal microbial farm dam communities. DF are degrees of freedom.

|  | **Numerator DF** | **Denominator DF** | ***F* value** | ***P* value** |
| --- | --- | --- | --- | --- |
| 1. **Bacteria** |  |  |  |  |
| Treatment × sampling site | 2 | 27 | 3.53 | 0.001 |
| Treatment × sample type | 1 | 27 | 4.68 | 0.001 |
| 1. **Archaea** |  |  |  |  |
| Treatment × sampling site | 2 | 27 | 3.86 | 0.001 |
| Treatment × sample type | 1 | 27 | 2.49 | 0.002 |

**Table S5:** Outcome of linear models testing for the effects of sampling site, treatment (fenced or unfenced farm dam), and sample type (water or sediment) on the relative abundances of (A) bacterial and (B) archaeal genera. DF are degrees of freedom.

| **Type** | **Family & Genus** | **Numerator DF** | **Denominator DF** | ***F*-value** | ***P*-value** |  |
| --- | --- | --- | --- | --- | --- | --- |
| 1. **Bacteria** | | | | | | |
| Methanotrophy | **Beijerinckiaceae – alphaI cluster** |  |  |  |  |  |
|  | Intercept | 1 | 24 | 22.97 | 0.0001 |  |
|  | Site | 2 | 24 | 10.38 | 0.0006 |  |
|  | Treatment | 1 | 24 | 5.49 | 0.03 |  |
|  | Sample type | 1 | 24 | 7.73 | 0.01 |  |
|  | Site × treatment | 2 | 24 | 8.16 | 0.002 |  |
|  | **Beijerinckiaceae – *Methylocystis*** |  |  |  |  |  |
|  | Intercept | 1 | 25 | 506.8 | <0.0001 |  |
|  | Site | 2 | 25 | 6.69 | 0.005 |  |
|  | Treatment | 1 | 25 | 4.62 | 0.04 |  |
|  | Sample type | 1 | 25 | 139.02 | <0.0001 |  |
|  | Site × treatment | 2 | 25 | 8.02 | 0.002 |  |
|  | **Beijerinckiaceae – *Methylorosula*** |  |  |  |  |  |
|  | Intercept | 1 | 11 | 0.3 | 0.59 |  |
|  | Site (Yarrawalla not included) | 1 | 11 | 2.13 | 0.17 |  |
|  | Treatment | 1 | 11 | 24.91 | 0.0004 |  |
|  | Sample type | 1 | 11 | 5.85 | 0.03 |  |
|  | **Methylacidiphilaceae – uncultured** |  |  |  |  |  |
|  | Intercept | 1 | 11 | 32.86 | 0.0001 |  |
|  | Site | 2 | 11 | 11.03 | 0.002 |  |
|  | Treatment | 1 | 11 | 2.51 | 0.14 |  |
|  | Sample type | 1 | 11 | 0.26 | 0.62 |  |
|  | Site × treatment | 2 | 11 | 21.07 | 0.0002 |  |
|  | **Methylococcaceae – *Methylocaldum*** |  |  |  |  |  |
|  | Intercept | 1 | 12 | 2.08 | 0.18 |  |
|  | Site (Buttermans not included) | 1 | 12 | 52.98 | <0.0001 |  |
|  | Treatment | 1 | 12 | 0.61 | 0.45 |  |
|  | Sample type | 1 | 12 | 4.93 | 0.05 |  |
|  | Site × treatment | 2 | 12 | 41.91 | <0.0001 |  |
|  | **Methylococcaceae – *Methyloparacoccus*** |  |  |  |  |  |
|  | Intercept | 1 | 17 | 2.33 | 0.15 |  |
|  | Site | 2 | 17 | 46.54 | <0.0001 |  |
|  | Treatment | 1 | 17 | 9.45 | 0.007 |  |
|  | Sample type | 1 | 17 | 16.52 | 0.0008 |  |
|  | Site × treatment | 2 | 17 | 28.56 | <0.0001 |  |
|  | **Methylococcaceae – uncultured** |  |  |  |  |  |
|  | Intercept | 1 | 39 | 66.99 | <0.0001 |  |
|  | Site | 2 | 39 | 2.89 | 0.07 |  |
|  | Treatment | 1 | 39 | 1.77 | 0.19 |  |
|  | Sample type | 1 | 39 | 4.2 | 0.04 |  |
|  | Site × treatment | 2 | 39 | 3.99 | 0.03 |  |
|  | **Methylomonadaceae – *Crenothrix*** |  |  |  |  |  |
|  | Intercept | 1 | 7 | 0.44 | 0.53 |  |
|  | Site | 2 | 7 | 1.15 | 0.37 |  |
|  | Treatment | 1 | 7 | 26.03 | 0.001 |  |
|  | Sample type | 1 | 7 | 0.006 | 0.94 |  |
|  | Site × treatment | 2 | 7 | 7.77 | 0.02 |  |
|  | **Methylomonadaceae – uncultured** |  |  |  |  |  |
|  | Intercept | 1 | 25 | 0.9 | 0.35 |  |
|  | Site | 2 | 25 | 0.65 | 0.53 |  |
|  | Treatment | 1 | 25 | 0.002 | 0.96 |  |
|  | Sample type | 1 | 25 | 0.03 | 0.89 |  |
|  | Site × treatment | 2 | 25 | 5.94 | 0.008 |  |
| Denitrification | **Burkholderiaceae – Burkholderia-Caballeronia-Paraburkholderia** |  |  |  |  |  |
|  | Intercept | 1 | 7 | 788.08 | <0.0001 |  |
|  | Site | 2 | 7 | 4.87 | 0.05 |  |
|  | Treatment | 1 | 7 | 26.82 | 0.001 |  |
|  | **Comamonadaceae – *Limnohabitans*** |  |  |  |  |  |
|  | Intercept | 1 | 12 | 1550.35 | <0.0001 |  |
|  | Site | 2 | 12 | 420.86 | <0.0001 |  |
|  | Treatment | 1 | 12 | 181.11 | <0.0001 |  |
|  | Sample type | 1 | 12 | 291.77 | <0.0001 |  |
|  | Site × treatment | 2 | 12 | 185.02 | <0.0001 |  |
|  | **Comamonadaceae – *Rhodoferax*** |  |  |  |  |  |
|  | Intercept | 1 | 19 | 275.8 | <0.0001 |  |
|  | Site | 2 | 19 | 1.11 | 0.35 |  |
|  | Treatment | 1 | 19 | 8.68 | 0.008 |  |
|  | Sample type | 1 | 19 | 181.34 | <0.0001 |  |
|  | **Nocardiaceae – *Rhodococcus*** |  |  |  |  |  |
|  | Intercept | 1 | 10 | 111.41 | <0.0001 |  |
|  | Site (Dixons not included) | 1 | 10 | 2.04 | 0.18 |  |
|  | Treatment | 1 | 10 | 11.63 | 0.007 |  |
|  | Sample type | 1 | 10 | 26.66 | 0.0004 |  |
|  | **Pseudomonadaceae – *Pseudomonas*** |  |  |  |  |  |
|  | Intercept | 1 | 9 | 228.55 | <0.0001 |  |
|  | Site | 2 | 9 | 0.54 | 0.6 |  |
|  | Treatment | 1 | 9 | 0.002 | 0.97 |  |
|  | Sample type | 1 | 9 | 0.33 | 0.58 |  |
|  | **Steroidobacteraceae – uncultured** |  |  |  |  |  |
|  | Intercept | 1 | 26 | 133.67 | <0.0001 |  |
|  | Site | 2 | 26 | 3.07 | 0.06 |  |
|  | Treatment | 1 | 26 | 0.25 | 0.62 |  |
|  | Sample type | 1 | 26 | 1.99 | 0.17 |  |
|  | **Xanthobacteraceae – *Bradyrhizobium*** |  |  |  |  |  |
|  | Intercept | 1 | 9 | 714.79 | <0.0001 |  |
|  | Site (Yarrawalla not included) | 1 | 9 | 1.51 | 0.25 |  |
|  | Treatment | 1 | 9 | 0.17 | 0.69 |  |
| Nitrification | **Nitrosomonadaceae – Ellin6067** |  |  |  |  |  |
|  | Intercept | 1 | 16 | 268.38 | <0.0001 |  |
|  | Site | 2 | 16 | 0.56 | 0.58 |  |
|  | Treatment | 1 | 16 | 1.72 | 0.21 |  |
|  | Sample type | 1 | 16 | 27.46 | 0.0001 |  |
|  | **Nitrosomonadaceae – MND1** |  |  |  |  |  |
|  | Intercept | 1 | 10 | 4.41 | 0.06 |  |
|  | Site (Yarrawalla not included) | 1 | 10 | 4.92 | 0.05 |  |
|  | Treatment | 1 | 10 | 5.89 | 0.04 |  |
|  | Sample type | 1 | 10 | 4.85 | 0.05 |  |
| 1. **Archaea** | | | | | | |
| ANME | **Methanoperedenaceae – *Candidatus* Methanoperedens** |  |  |  |  |  |
|  | Intercept | 1 | 12 | 48.23 | <0.0001 |  |
|  | Site | 2 | 12 | 2.76 | 0.1 |  |
|  | Treatment | 1 | 12 | 0.66 | 0.43 |  |
|  | Sample type | 1 | 12 | 0.55 | 0.47 |  |
|  | Site × treatment | 2 | 12 | 9.22 | 0.004 |  |
| Methanogenesis | **Methanocellaceae – *Methanocella*** |  |  |  |  |  |
|  | Intercept | 1 | 19 | 52.32 | <0.0001 |  |
|  | Site | 2 | 19 | 6.61 | 0.007 |  |
|  | Treatment | 1 | 19 | 6.68 | 0.02 |  |
|  | Sample type | 1 | 19 | 1.49 | 0.24 |  |
|  | Site × treatment | 2 | 19 | 3.9 | 0.04 |  |
|  | **Methanocellaceae – Rice Cluster I** |  |  |  |  |  |
|  | Intercept | 1 | 22 | 155.08 | <0.0001 |  |
|  | Site | 2 | 22 | 7.69 | 0.003 |  |
|  | Treatment | 1 | 22 | 0.65 | 0.43 |  |
|  | Sample type | 1 | 22 | 16.49 | 0.0005 |  |
|  | **Methanomassiliicoccaceae – uncultured** |  |  |  |  |  |
|  | Intercept | 1 | 23 | 17.7 | 0.0003 |  |
|  | Site | 2 | 23 | 13.47 | 0.0001 |  |
|  | Treatment | 1 | 23 | 2.24 | 0.15 |  |
|  | Sample type | 1 | 23 | 0.62 | 0.44 |  |
|  | Site × treatment | 2 | 23 | 7.94 | 0.002 |  |
|  | **Methanomethylophilaceae – uncultured** |  |  |  |  |  |
|  | Intercept | 1 | 5 | 5.38 | 0.07 |  |
|  | Site (Dixons not included) | 1 | 5 | 11.98 | 0.02 |  |
|  | Treatment | 1 | 5 | 3.49 | 0.12 |  |
|  | Sample type | 1 | 5 | 19.58 | 0.007 |  |
|  | Site × treatment | 1 | 5 | 15.99 | 0.01 |  |
|  | **Methanoregulaceae – *Methanolinea*** |  |  |  |  |  |
|  | Intercept | 1 | 14 | 979.78 | <0.0001 |  |
|  | Site (Dixons not included) | 1 | 14 | 18.64 | 0.0007 |  |
|  | Treatment | 1 | 14 | 15.15 | 0.002 |  |
|  | Sample type | 1 | 14 | 2.34 | 0.15 |  |
|  | **Methanoregulaceae – *Methanoregula*** |  |  |  |  |  |
|  | Intercept | 1 | 23 | 9.48 | 0.005 |  |
|  | Site | 2 | 23 | 10.31 | 0.0006 |  |
|  | Treatment | 1 | 23 | 0.04 | 0.85 |  |
|  | Sample type | 1 | 23 | 1.54 | 0.23 |  |
|  | Site × treatment | 2 | 23 | 3.66 | 0.04 |  |
|  | **Methanosaetaceae – *Methanosaeta*** |  |  |  |  |  |
|  | Intercept | 1 | 20 | 67.92 | <0.0001 |  |
|  | Site | 2 | 20 | 39.88 | <0.0001 |  |
|  | Treatment | 1 | 20 | 6.77 | 0.02 |  |
|  | Sample type | 1 | 20 | 9.36 | 0.006 |  |
|  | **Methanosarcinaceae – *Methanosarcina*** |  |  |  |  |  |
|  | Intercept | 1 | 24 | 66.45 | <0.0001 |  |
|  | Site | 2 | 24 | 3.24 | 0.06 |  |
|  | Treatment | 1 | 24 | 4.64 | 0.04 |  |
|  | Sample type | 1 | 24 | 30.09 | <0.0001 |  |
|  | **Methanospirillaceae – *Methanospirillum*** |  |  |  |  |  |
|  | Intercept | 1 | 11 | 248.76 | <0.0001 |  |
|  | Site (Dixons not included) | 1 | 11 | 1.76 | 0.21 |  |
|  | Treatment | 1 | 11 | 1.2 | 0.29 |  |
|  | Sample type | 1 | 11 | 5.11 | 0.04 |  |
|  | **Uncultured (Methanomicrobiales)** |  |  |  |  |  |
|  | Intercept | 1 | 25 | 30.11 | <0.0001 |  |
|  | Site | 2 | 25 | 6.66 | 0.005 |  |
|  | Treatment | 1 | 25 | 1.26 | 0.27 |  |
|  | Sample type | 1 | 25 | 7.73 | 0.01 |  |
|  | Site × treatment | 2 | 25 | 2.61 | 0.09 |  |
| Nitrification | **Nirosopumilaceae – uncultured** |  |  |  |  |  |
|  | Intercept | 1 | 14 | 8.46 | 0.01 |  |
|  | Site | 2 | 14 | 2.21 | 0.15 |  |
|  | Treatment | 1 | 14 | 0.01 | 0.93 |  |
|  | Sample type | 1 | 14 | 0.16 | 0.69 |  |
|  | **Nitrososphaeraceae – *Candidatus* Nitrocosmicus** |  |  |  |  |  |
|  | Intercept | 1 | 22 | 117.93 | <0.0001 |  |
|  | Site | 2 | 22 | 0.94 | 0.41 |  |
|  | Treatment | 1 | 22 | 8.94 | 0.007 |  |
|  | Sample type | 1 | 22 | 11.29 | 0.003 |  |
|  | **Nitrososphaeraceae – uncultured** |  |  |  |  |  |
|  | Intercept | 1 | 23 | 186.51 | <0.0001 |  |
|  | Site | 2 | 23 | 8.95 | 0.001 |  |
|  | Treatment | 1 | 23 | 3.56 | 0.07 |  |
|  | Sample type | 1 | 23 | 0.58 | 0.45 |  |
|  | **Nitrosotaleaceae – *Candidatus* Nitrosotalea** |  |  |  |  |  |
|  | Intercept | 1 | 16 | 0.003 | 0.96 |  |
|  | Site | 2 | 16 | 0.29 | 0.75 |  |
|  | Treatment | 1 | 16 | 120.31 | <0.0001 |  |
|  | Sample type | 1 | 16 | 1.81 | 0.19 |  |
|  | Site × treatment | 2 | 16 | 25.5 | <0.0001 |  |

**Table S6:** Outcome of linear models testing for the effects of treatment (fenced or unfenced farm dam), site, and sample type (sediment or water) on gene abundances of (A) *mcrA*, (B) *r-mcrA*, (C) *mmoX*, (D) *pmoA*, (E) *nirS*, (F) *nirK*, (G) *norB*, (H) *narG,* (I) *napA*, (J) *amoA*, (K) *nxrA*, and (L) *nosZ type I* and (K) *nosZ type II*. DF are degrees of freedom.

|  | **Numerator DF** | **Denominator DF** | ***F* value** | ***P* value** |
| --- | --- | --- | --- | --- |
| 1. ***mcrA*** |  |  |  |  |
| Intercept | 1 | 7 | 45.61 | 0.0003 |
| Treatment | 1 | 7 | 4.37 | 0.07 |
| Site | 2 | 7 | 2.79 | 0.13 |
| Sample type | 1 | 7 | 252.87 | <0.0001 |
| 1. ***r-mcrA*** |  |  |  |  |
| Intercept | 1 | 7 | 559038.2 | <0.0001 |
| Treatment | 1 | 7 | 1.5 | 0.26 |
| Site | 2 | 7 | 1.4 | 0.3 |
| Sample type | 1 | 7 | 1.1 | 0.32 |
| 1. ***mmoX*** |  |  |  |  |
| Intercept | 1 | 7 | 16.41 | 0.005 |
| Treatment | 1 | 7 | 4.08 | 0.08 |
| Site | 2 | 7 | 2 | 0.21 |
| Sample type | 1 | 7 | 5.79 | 0.04 |
| 1. ***pmoA*** |  |  |  |  |
| Intercept | 1 | 7 | 27.89 | 0.001 |
| Treatment | 1 | 7 | 18.17 | 0.004 |
| Site | 2 | 7 | 1.56 | 0.28 |
| Sample type | 1 | 7 | 161.52 | <0.0001 |
| 1. ***nirS*** |  |  |  |  |
| Intercept | 1 | 5 | 7.16 | 0.04 |
| Treatment | 1 | 5 | 12.17 | 0.02 |
| Site | 2 | 5 | 0.03 | 0.97 |
| Sample type | 1 | 5 | 0.42 | 0.54 |
| Site × treatment | 2 | 5 | 5.43 | 0.05 |
| 1. ***nirK*** |  |  |  |  |
| Intercept | 1 | 6 | 122.89 | <0.0001 |
| Treatment | 1 | 6 | 4.73 | 0.07 |
| Site | 2 | 6 | 5.58 | 0.04 |
| Sample type | 1 | 6 | 29.06 | 0.002 |
| Treatment × Sample type | 1 | 6 | 6.59 | 0.04 |
| 1. ***norB*** |  |  |  |  |
| Intercept | 1 | 7 | 88.36 | <0.0001 |
| Treatment | 1 | 7 | 3.82 | 0.09 |
| Site | 2 | 7 | 1.83 | 0.23 |
| Sample type | 1 | 7 | 78.86 | <0.0001 |
| 1. ***narG*** |  |  |  |  |
| Intercept | 1 | 7 | 86.7 | <0.0001 |
| Treatment | 1 | 7 | 28.71 | 0.001 |
| Site | 2 | 7 | 2.98 | 0.12 |
| Sample type | 1 | 7 | 274.44 | <0.0001 |
| 1. ***napA*** |  |  |  |  |
| Intercept | 1 | 7 | 226.98 | <0.0001 |
| Treatment | 1 | 7 | 0.53 | 0.49 |
| Site | 2 | 7 | 0.16 | 0.86 |
| Sample type | 1 | 7 | 252.05 | <0.0001 |
| 1. ***amoA*** |  |  |  |  |
| Intercept | 1 | 7 | 23.74 | 0.002 |
| Treatment | 1 | 7 | 1.01 | 0.35 |
| Site | 2 | 7 | 0.11 | 0.89 |
| Sample type | 1 | 7 | 13.09 | 0.009 |
| 1. ***nxrA*** |  |  |  |  |
| Intercept | 1 | 7 | 71.37 | 0.0001 |
| Treatment | 1 | 7 | 0.04 | 0.86 |
| Site | 2 | 7 | 0.47 | 0.65 |
| Sample type | 1 | 7 | 67.69 | 0.0001 |
| 1. ***nosZ type I*** |  |  |  |  |
| Intercept | 1 | 7 | 44.25 | 0.0003 |
| Treatment | 1 | 7 | 6.6 | 0.04 |
| Site | 2 | 7 | 0.49 | 0.63 |
| Sample type | 1 | 7 | 58.08 | 0.0001 |
| 1. ***nosZ type II*** |  |  |  |  |
| Intercept | 1 | 7 | 509.58 | <0.0001 |
| Treatment | 1 | 7 | 13.88 | 0.01 |
| Site | 2 | 7 | 1.75 | 0.27 |
| Sample type | 1 | 7 | 179.69 | <0.0001 |
| Site × treatment | 2 | 7 | 8.79 | 0.02 |

**Table S7:** Average abundances (copy numbers per organism) of genes mediating CH_4_ and N_2_O fluxes from fenced and unfenced dams. *P* values indicate the significance level of the effect of treatment (unfenced vs. unfenced) on average gene abundance, unless otherwise specified.

| **Gene** | **Function** | **Site** | **Fenced dams** | **Unfenced dams** | ***P* value** |
| --- | --- | --- | --- | --- | --- |
| *mcrA* | Methanogenesis (archaea) | Buttermans | 0.008 ± 0.002 | 0.006 ± 0.002 | 0.07 |
|  |  | Dixons | 0.003 ± 0.004 | 0.001 ± 0.004 |  |
|  |  | Yarrawalla | 0.012 ± 0.0008 | 0.009 ± 0.0008 |  |
| *r-mcrA* | Methanotrophy (archaea) | Buttermans | 0.0003 ± 0.0002 | 0.0004 ± 0.0002 | 0.26 |
|  |  | Dixons | 0.0001 ± 0. 0002 | 0.0002 ± 0.0002 |  |
|  |  | Yarrawalla | 0.0001 ± 0.0002 | 0.0002 ± 0.0002 |  |
| *mmoX* | Methanotrophy (bacteria) | Buttermans | 0.0004 ± 0.0001 | 0.0001 ± 0.0001 | 0.08 |
|  |  | Dixons | 0.0006 ± 0.0001 | 0.0003 ± 0.0001 |  |
|  |  | Yarrawalla | 0.001 ± 0.0005 | 0.0009 ± 0.0005 |  |
| *pmoA* | Methanotrophy (bacteria) | Buttermans | 0.02 ± 0.001 | 0.01 ± 0.001 | 0.004 |
|  |  | Dixons | 0.03 ± 0.01 | 0.02 ± 0.01 |  |
|  |  | Yarrawalla | 0.05 ± 0.02 | 0.04 ± 0.02 |  |
| *nirS* | Denitrification-associated N_2_O production (bacteria) | Buttermans | 0.01 ± 0.004 | 0.03 ± 0.004 | 0.05 (site × treatment) |
|  |  | Dixons | 0.01 ± 0.004 | 0.01 ± 0.004 |  |
|  |  | Yarrawalla | 0.01 ± 0.004 | 0.03 ± 0.004 |  |
| *nirK* | Denitrification-associated N_2_O production (bacteria) | Buttermans | Water: 0.05 ± 0.006  Sediment: 0.13 ± 0.02 | Water: 0.05 ± 0.005  Sediment: 0.21 ± 0.04 | 0.04 (treatment × sample type) |
|  |  | Dixons | Water: 0.03 ± 0.004  Sediment: 0.09 ± 0.02 | Water: 0.03 ± 0.003  Sediment: 0.14 ± 0.03 |  |
|  |  | Yarrawalla | Water: 0.03 ± 0.004  Sediment: 0.08 ± 0.01 | Water: 0.02 ± 0.003  Sediment: 0.13 ± 0.02 |  |
| *norB* | Denitrification-associated N_2_O production (bacteria) | Buttermans | 0.13 ± 0.02 | 0.15 ± 0.02 | 0.09 |
|  |  | Dixons | 0.11 ± 0.02 | 0.13 ± 0.02 |  |
|  |  | Yarrawalla | 0.13 ± 0.02 | 0.16 ± 0.02 |  |
| *narG* | Denitrification-associated N_2_O production (bacteria) | Buttermans | 0.11 ± 0.02 | 0.16 ± 0.02 | 0.001 |
|  |  | Dixons | 0.08 ± 0.01 | 0.13 ± 0.01 |  |
|  |  | Yarrawalla | 0.12 ± 0.01 | 0.17 ± 0.01 |  |
| *napA* | Denitrification-associated N_2_O production (bacteria) | Buttermans | 0.03 ± 0.003 | 0.03 ± 0.003 | 0.49 |
|  |  | Dixons | 0.03 ± 0.002 | 0.03 ± 0.002 |  |
|  |  | Yarrawalla | 0.03 ± 0.009 | 0.03 ± 0.009 |  |
| *amoA* | Nitrification-associated N_2_O production (bacteria) | Buttermans | 0.001 ± 0.0004 | 0.001 ± 0.0004 | 0.35 |
|  |  | Dixons | 0.001 ± 0.0008 | 0.001 ± 0.0008 |  |
|  |  | Yarrawalla | 0.001 ± 0.0008 | 0.0005 ± 0.0008 |  |
| *nxrA* | Nitrification-associated N_2_O production (bacteria) | Buttermans | 0.006 ± 0.0007 | 0.006 ± 0.0007 | 0.85 |
|  |  | Dixons | 0.006 ± 0.0007 | 0.006 ± 0.0007 |  |
|  |  | Yarrawalla | 0.006 ± 0.0007 | 0.006 ± 0.0007 |  |
| *nosZ type I* | N_2_O reduction (bacteria) | Buttermans | 0.03 ± 0.006 | 0.04 ± 0.006 | 0.04 |
|  |  | Dixons | 0.02 ± 0.006 | 0.03 ± 0.006 |  |
|  |  | Yarrawalla | 0.02 ± 0.006 | 0.04 ± 0.006 |  |
| *nosZ type II* | N_2_O reduction (bacteria) | Buttermans | 0.03 ± 0.003 | 0.05 ± 0.006 | 0.02 (site × treatment) |
|  |  | Dixons | 0.03 ± 0.003 | 0.03 ± 0.003 |  |
|  |  | Yarrawalla | 0.04 ± 0.005 | 0.08 ± 0.009 |  |

**Table S10:** Outcome of linear models testing for the effects of treatment (fenced or unfenced farm dam), site, and type (*Methylocystis* sp. or *CAIVXW01* sp.) on the relative abundances of the previously uncharacterised methanotrophs. DF are degrees of freedom.

|  | **Numerator DF** | **Denominator DF** | ***F* value** | ***P* value** |
| --- | --- | --- | --- | --- |
| Intercept | 1 | 7 | 0.45 | 0.52 |
| Type | 1 | 7 | 3.71 | 0.09 |
| Treatment | 1 | 7 | 1.51 | 0.26 |
| Site | 2 | 7 | 4.18 | 0.06 |
